# At Least Two Genes Encode Many Variants of Irak3 in Rainbow Trout, but Neither the Full-Length Factor Nor Its Variants Interfere Directly With the TLR-Mediated Stimulation of Inflammation

**DOI:** 10.3389/fimmu.2019.02246

**Published:** 2019-09-20

**Authors:** Alexander Rebl, Henrike Rebl, Marieke Verleih, Stephanie Haupt, Judith M. Köbis, Tom Goldammer, Hans-Martin Seyfert

**Affiliations:** ^1^Fish Genetics Unit, Leibniz Institute for Farm Animal Biology (FBN), Institute of Genome Biology, Dummerstorf, Germany; ^2^Department of Cell Biology, Rostock University Medical Center, Rostock, Germany

**Keywords:** CHSE-214, GFP expression plasmid, IRAK-3, salmonid fishes, Toll-like receptor signaling

## Abstract

The interleukin-1-receptor-associated kinase 3 (IRAK3) is known in mammals as a negative feedback regulator of NF-κB-mediated innate-immune mechanisms. Our RNA-seq experiments revealed a prototypic 1920-nt sequence encoding *irak3* from rainbow trout (*Oncorhynchus mykiss*), as well as 20 variants that vary in length and nucleotide composition. Based on the DNA-sequence information from two closely related *irak3* genes from rainbow trout and an *irak3-*sequence fragment from Atlantic salmon retrieved from public databases, we elucidated the underlying genetic causes for this striking *irak3* diversity. Infecting rainbow trout with a lethal dose of *Aeromonas salmonicida* enhanced the expression of all variants in the liver, head kidney, and peripheral blood leucocytes. We analyzed the functional impact of the full-length factor and selected structural variants by overexpressing them in mammalian HEK-293 cells. The full-length factor enhanced the basal activity of NF-κB, but did not dampen the TLR2-signaling-induced levels of NF-κB activation. Increasing the basal NF-κB-activity through Irak3 apparently does not involve its C-terminal domain. However, more severely truncated factors had only a minor impact on the activity of NF-κB. The TLR2-mediated stimulation did not alter the spatial distribution of Irak3 inside the cells. In salmonid CHSE-214 cells, we observed that the Irak3-splice variant that prominently expresses the C-terminal domain significantly quenched the stimulation-dependent production of interleukin-1β and interleukin-8, but not the production of other immune regulators. We conclude that the different gene and splice variants of Irak3 from trout play distinct roles in the activation of immune-regulatory mechanisms.

## Introduction

The immune system of vertebrates enables rapid, very efficient, and sustainable defense against microorganisms and viruses. Shortly after the detection of pathogens, amplifier mechanisms boost various destructive activities, which may also be directed against the host itself. To avoid such pathophysiological conditions, the immune system is tightly regulated by a vast number of inhibitory factors (1). One of the factors that counteracts pro-inflammatory processes is the interleukin-1-receptor-associated kinase 3 (IRAK3) alias IRAK-M. In general, all four IRAK-family members (IRAK1-4) are composed of four defined regions: an N-terminal death domain, the ProST region, which is rich in proline, serine, and threonine residues, and a kinase domain adjacent to the C-terminal region (2–5). In contrast to IRAK3, the IRAK-family members−1,−2, and−4 positively regulate inflammatory processes. While mammalian species express all four IRAK paralogs, genomes of several non-mammalian vertebrates feature only one or two IRAK paralogs (2). An IRAK3 ortholog has, for instance, been lost in sauropsidia, amphibians (2, 6), and several fish species.

The IRAK proteins are constituents of the multimeric signaling complex “myddosome” (7) that transfers the pathogen-related signals from toll-like receptors (TLRs) (8, 9) to downstream factors. These include TRAF6 (“tumor necrosis factor alpha”-receptor–associated factor 6), which is crucially involved in releasing active NF-κB/Rel proteins from their inhibitors (10). These key activators of transcription control hundreds of pro-inflammatory immune genes (11), such as the cytokine genes interleukin-1β (*IL1B*) and interleukin-8 (*CXCL8*).

The structural basis for the different regulatory roles of the four IRAK proteins lies in an amino-acid exchange. While an aspartic-acid residue is vital for the catalytic activity of the kinase domain in the human IRAK1 and−4 factors (12), this position is substituted with an asparagine residue in human IRAK2 and a serine residue in human IRAK3 (Ser-293) (3, 12). The latter exchange knocks down kinase activity, so IRAK3 contains only a “pseudokinase domain.”

The interleukin-1-receptor-associated kinase 3 (IRAK3) is thought to block the TLR-dependent activation of NF-κB proteins and MAP kinases (13, 14) and contribute to establishing tolerance to microbial components such as endotoxins (15–18). Two mechanisms have been postulated in this regard. Lyn-Kew suggested that IRAK3 allows the epigenetic remodeling of chromatin by affecting the expression of histone deacetylase 2, which silences the synthesis of pro-inflammatory effectors (19). Zhou and co-workers proved in mice that IRAK3 may interact with IRAK4 to form the so-called “IRAK-M myddosome” (5). This specific variant of the complex mediates a second-wave of NF-κB activation that induces the expression of genes encoding anti-inflammatory molecules like SOCS1 (suppressor of cytokine signaling 1), INPP5D (inositol polyphosphate-5-phosphatase D alias SHIP1), TNFAIP3 (TNF alpha-induced protein 3 alias A20), and NFKBIA (NF- κB inhibitor alpha alias IκBα) (5, 17). Eventually, these counterbalancing factors prevent the progression of a “cytokine storm” and contribute to the resolution of inflammation (20).

Two components of the putative myddosome of rainbow trout (*Oncorhynchus mykiss*) have already been characterized by our group, the TLR adapter *myd88* and its binding partner *irak4* (21–23). So far, *irak1* has not been investigated in trout, but has in other fish species (24, 25), and *irak2* has been lost in fish (26). The first mention of *irak3* as a promising marker for the early immune response of zebrafish is found in a report by Stockhammer et al. (27), but it took another 9 years until the first Irak3 factor was structurally und functionally characterized in a (serranid) fish species (28).

Meanwhile, we also began analyzing the structure and function of Irak3 from rainbow trout, but we encountered several problems. First and above all, we consistently obtained *irak3* amplificates with various *irak3*-specific PCR primers, which all showed atypical shoulder peaks in melting-curve analysis, indicating the presence of various isoforms. We wondered (i) how many Irak3 variants are expressed in trout, (ii) how would they differ structurally, and (iii) what physiological effects the different variants might cause during the immune response of trout. The present report characterizes more than 20 *irak3*-mRNA isotypes including their splice variants from rainbow trout. We expressed four structurally very different *irak3* transcripts in two cell models and quantified their potential to interfere with immune-activating mechanisms.

## Materials and Methods

### Identification of Irak3-Encoding Sequences in RNA-Seq Datasets From Rainbow Trout

At the outset of our investigations of an IRAK3 ortholog in salmonid fish, no full-length cDNA sequence for any teleostean ortholog was known. Therefore, we performed a BLAST search against the next-generation RNA-sequence reads of rainbow trout *O. mykiss*, which had been generated in our institute using the Illumina RNA-seq technology. The procedure is only briefly presented below, since a more detailed presentation of the experiment has already been published elsewhere (29). Total RNA was extracted from six tissues (gills, head kidney, heart, liver, spleen, white muscle) of rainbow trout. Trout were 10–11 months in age with an average weight of 333.0 ± 54.9 g. Library constructions followed the TruSeq RNA Sample Preparation v2 Guide supplied with the TruSeq RNA Sample Prep Kit v2 (Illumina) with minor modifications. Three micrograms of total RNA were purified, fragmented, and then used as template for cDNA synthesis. Subsequently, Illumina paired-end adapters were ligated to the 3′-adenylated cDNA ends. After determining their DNA concentrations, the libraries of each tissue from eight individuals per strain, marked with distinct adapters, were loaded into separate Illumina-flow cells. Thirty-six sequencing cycles were conducted on an Illumina Genome Analyzer using the Multiplex Paired-End-Sequencing kits (Illumina). The resulting short-sequence reads were filtered for quality by our in-house Linux program FILTRIX (unpublished). The filter steps included the removal of adapter sequences to trim away low-quality regions and merge overlapping reads. Based on this primary-data analysis comprising 332 million high-quality sequencing reads of the trout transcriptome, we used the bioinformatics software Unipro UGENE v1.16 (30) to search for *irak3* reads.

### Isolation and Cloning of Multiple *Irak3*-Sequences From Rainbow Trout

To isolate the cDNA sequences that encode the entire open-reading frame (ORF) of *irak3* and its supposed variants, we reverse-transcribed total RNA extracted from various rainbow-trout tissues (see above) using a GeneRacer Kit (ThermoFisherScientific) and the SuperScript II Reverse Transcriptase (ThermoFisherScientific). Forward primers were placed on the 5′-UTR immediately upstream of the translational start codon and the reverse primers on the 3′-UTR, downstream adjacent to the translational stop codon (full-length, sense f1: 5′-TAAGGTTCCAGCTCAGTCGC-3′, antisense r1: 5′-GATTAGGATTCGGGAGGGCA-3′; truncated, sense f2: 5′-CGCAGTTGAGATGGACTCGTC-3′, antisense r2: 5′-ACATTGGTTTCCTTACCCTGTCA-3′). In a standard-PCR reaction using the *OneTaq* DNA Polymerase (New England Biolabs) and a pool of previously generated cDNAs as template, we retrieved fragments of up to ~2 kb.

In addition, we performed 3′-RACE experiments to elucidate the 3′-end sequence of the truncated *irak3* variants. To this end, we used the sense RACE-primer f1 5′-AAGCTGTCTGATTTTGGGACGG-3′ derived from the 5′-region of exon 9 (5′ upstream of the site where the coding region of the truncated *irak3* variant terminates) and a nested RACE-primer f2 5′-TGCCGGAGGAATACATACGATG-3′ located further downstream on the same exon. An oligo(d)T oligonucleotide served as antisense primer. All PCR amplicons were cloned into pGEM-T Easy vector (Promega) and sequenced using the MegaBACE capillary sequencer (GE Healthcare). Each nucleotide position was sequenced at least four times.

### *In-silico* Structural Characterization of Irak3 Variants

Global sequence alignments were performed using the EMBOSS/Stretcher tool (https://www.ebi.ac.uk/Tools/psa/emboss_stretcher/). Phylogenetic analyses were conducted using the Molecular Evolutionary Genetics Analysis package MEGA7 (31). We first clipped all *irak3* sequence files at position 994 in exon 9 (relative to the first coding nucleotide of variant a), which represents the common fragment of the truncated and full-length Irak3-encoding cDNAs and then subjected these to multiple alignment using the ClustalW algorithm. The dendrogram was reconstructed using the neighbor-joining method based on the maximum-likelihood method. The tree with the highest log likelihood (−1338.9) was chosen and optimized manually. The online tool Microsatellite Repeats Finder (http://insilico.ehu.es/mini_tools/microsatellites/) was used to identify repetitive elements in the genomic DNA. Functional motifs and domains of conceptually translated (Expert Protein Analysis System proteomics server, Swiss Institute of Bioinformatics; http://www.expasy.ch/) amino acid (aa) sequences were identified using the NCBI (National Center for Biotechnology Information; http://blast.ncbi.nlm.nih.gov/) algorithm in accordance with results from the search with SMART (Simple Modular Architecture Research Tool; http://smart.embl-heidelberg.de/). The three-dimensional (3D) structures of particular Irak3 variants were predicted by the Protein Homology/analogY Recognition Engine V 2.0 (Phyre^2^) (32) and visualized using OpenRasMol software (33).

### Establishment of *Irak3*-Expression Plasmids

We constructed the plasmids that express the full-length *irak3* factor (m) and its variants (a, g, h, k, l, and m″) from rainbow trout to analyze their ability to interfere with TLR-dependent NF-κB activation in *in-vitro* models. In the first step, we designed variant-specific oligonucleotide primers featuring restriction sites for *Hind*III and *EcoR*I at their 5′ and 3′ ends, respectively. Subsequently, a standard-PCR reaction using cDNA from rainbow trout as template was conducted using the common sense primer 5′-ACCCAAGCTTGATATGGACTCGTCTATGTACCTGTA-3′ and the antisense primer 5′-TCAGAATTCCTCCTGGGTGGTCATGGAGA-3′ specific for variants m and m″ or the antisense primer 5′-TCAGAATTCAAAGCATCGTATGTATTCCTCCG-3′ specific for variants a and l (restriction sites of all primers are underlined). The resulting amplicons were inserted into pGEM-T Easy Vector (Promega). This subclone was digested with *Hind*III and *EcoR*I at 37°C overnight. The retrieved fragments were finally inserted “in frame” into our expression vector 280, a modified (34) CMV-driven pKS(+) Bluescript plasmid (Promega).

To analyze the intracellular spatial distribution, we tagged the full-length Irak3 factor m and its variants a and m″ with fluorescent labeling. Then, we digested the *irak3-a, -m* and *-m*″ subclones with *Hind*III and *EcoR*I at 37°C overnight and inserted the retrieved fragments into the respective cloning sites of plasmid pAM505, which expresses the green fluorescent protein (GFP; **AF140578**) under the control of the CMV promoter.

All plasmids were prepared with endotoxin-free reagents (EndoFree Plasmid Maxi Kit; Qiagen) and sequenced to confirm their appropriate construction.

### Cell Culture, Transfection, and Luciferase Assay

The human embryonic kidney-cell line HEK-293 (ATTC) and the embryonic cell line CHSE-214 from Chinook salmon *Oncorhynchus tshawytscha* (Sigma-Aldrich) were cultured in EMEM medium (Sigma-Aldrich), supplemented with 10% fetal calf serum (PAN), 1% non-essential aa (Biochrom), and 2 mM L-glutamine (Biochrom). The two cell lines were cultured in humidified atmosphere containing 5% CO_2_, at either 37°C (HEK-293) or 20°C (CHSE-214). Prior to and during the stimulation experiments, both cell lines were proven to be free of mycoplasma contamination using the Lookout Mycoplasma PCR detection kit (Sigma-Aldrich).

Endotoxin-free preparations of the expression constructs were transfected into HEK-293 cells using Lipofectamin 2000 (ThermoFisherScientific). For these co-transfection assays, we used 50 ng of the ELAM-driven NF-κB-reporter vector and increasing concentrations (50, 500, 2,000 ng) of the respective *irak3*-expression constructs. Since the HEK-293 cells do not express most TLRs (35), 200 ng of the plasmid that expresses bovine TLR2 (34) were co-transfected. The total DNA concentration was adjusted to 4,100 ng/assay by adding the empty cloning vector. After transfection, the cells were distributed into six wells of a 24-well plate. Three wells were left as unstimulated controls. The other three wells were stimulated for 24 h with the TLR2 ligands Pam_2_CSK_4_ (10 ng/ml, Invivogen) and FSL-1 (100 ng/ml, Invivogen) or with heat-killed bacteria *Escherichia coli*, strain 1303 (30 μg/ml). The luciferase activity of HEK-293-cell lysates was measured with the Dual-Luciferase Reporter Assay System (Promega) with the Lumat LB 9501 luminometer (Berthold). Values were normalized against the protein concentration as determined using the NanoDrop 2000 photometer (ThermoFisherScientific). Every transfection was assayed in triplicate; each transfection experiment was performed at least twice.

CHSE-214 cells were transfected with 2,000 ng of the plasmid that expresses trout *irak3* variants a, l, m, and m″ using X-tremeGENE HP DNA Transfection Reagent (Roche/Sigma-Aldrich). Since CHSE-214 cells endogenously express Tlr3 and Tlr5 (36), we stimulated them for 3 or 6 h with a mixture of the TLR3 ligand poly(I:C) of low molecular weight (10 μg/ml, Invivogen) and the TLR5 ligand flagellin from *Salmonella typhimurium* (100 ng/ml, Invivogen). These incubation times and doses were determined in pre-tests. After the stimulation period, cells were harvested in ice-cold PBS for RNA isolation and the subsequent profiling of immune-gene expression.

### Confocal Microscopy and Determination of Cell Viability

HEK-293 cells were transfected with 1 μg of plasmid expressing the GFP-tagged factors. Optionally, transfected cells were stimulated for 30 min with 10 ng/ml Pam_2_CSK_4_ prior to observation with fluorescence microscopy using the confocal microscope LSM 780 (Carl Zeiss Microscopy), equipped with a 63 × oil-immersion DIC objective and an incubation chamber to maintain ~37°C and 5% CO_2_ during the microscopic investigations. Nuclei were stained by adding Hoechst 33342 dye (1 μg/ml, Sigma-Aldrich) to the medium for 30 min, immediately before the start of the observation. Quantification of the live/dead ratio of transfected cells was performed with the Nucleocounter3000 (Chemometec). Dead cells were stained using propidium iodide (PI, 1 μg/ml). The fluorescence microscopic observation of the HEK-293 cells overexpressing the full-length (m), truncated (a), or splice (m″) *irak3* variants were repeated at least twice with different plasmid preparations.

### Quantitative Real-Time Expression Analyses of *in vivo* and *in vitro* Samples

The correlation between IRAK3-transcript and protein levels has been proven (16). For expression profiling, we designed a panel of transcripts that encode inflammatory and inhibitory factors from trout, including the primers that discriminate between different *irak3* transcripts (Table 1). To determine the abundance of the entirety of *irak3* transcripts, we designed oligonucleotide primers specific to exon 1 of the *irak3* gene (covering the region between positions 2 and 95 on the coding sequence of all unspliced variants) sharing perfect sequence identity across the multiple variants. A second pair was placed on exons 10 and 11 (positions 1,136 to 1,298 on the coding sequence of factors m and n), which are absent in all truncated variants, to quantify the abundance of the full-length transcripts.

**Table 1:** Gene-specific primers used for RT-qPCR assays.

**Gene symbol**	**Accession code**	**Sense primer (5^**′**^-3^**′**^)**	**Antisense primer (5^**′**^-3^**′**^)**	**Primer efficiency (%)**	**Fragment length (bp)**
*irak3* (all variants)	LN828695	TGGACTCGTCTATGTACCTGTAT	GCGCCATCCGAGGCTGTCAT	99.3	95
*irak3* (full-length)	LR031305	CCCTGCTGAGAAACGTCCTAC	GGTCTGTTGCGTGGTCGTGTA	99.2	162
*il1rl1*(*st2*)	NM_001281424	GATCTGAGGATGGGAAGGTCTA	CTGCCGTGGATGAAGAGTCTAA	98.0	154
*tollip*	AJ878917	GTGGTACAGCCTCAGTGGCA	GCTGATAGACTGTGGGCATGA	106.3	131
*inpp5d* (*ship1*)	XM_021615844	CAAACCCTCTGACCAGGACCC	GCCTGGGCGGGACTTCATGG	93.8	220
*tnfaip3 (a20*)	XM_021576327	CTTCAAGTTCTGCGTTTGTACACA	CTTGACCGCTTTCAGCAGGTTA	98.4	139
*usp4*	XM_021609985	GACGAGTGGTTTCTGATTGATAG	TTGTCAACAGGGCCGGGATAG	101.0	119
*serping1* (*c1inh*)	NM_001124379	AAGGAATGACGAACGGCAAACG	TCAGCTGTCTCACAGTAGTACAT	100.9	169
*c4bp*	XM_021564396	GAAGTGATGAACGGCCGATATAA	ACCACTTCACATACAGGAACTCT	99.6	152
*cfi*	XM_021593383	ACCCAGTGTTTGCAAGAGAACC	CAGTTGGCGATCAGAGAGACG	101.1	167
*cfh*	XR_002473365	GCTGGACCAAGACACTTGGC	CACCAACCCCCGGTAGAGG	102.1	166
*cd59*	XM_021606996	GATTGAGTGGGCAAAGTATTGTAT	AGCAATGTTATGTAACAGGGTATG	98.1	167
*a2m*	XM_021582312	GGGAGGAAGGATGAGATGAGTA	GGTCCTGAAGCTCCACTGTTAG	101.1	184
*nfkbia-1*	NM_001124368	AACCCTGGAGGAAAACAGTGAC	CGCCGTCTGTCTCTGATTGTTC	100.6	153
*nfkbia-2*	XM_021574049	TGAAGTTGTCGCCAGTGAGCTC	GGCTCATTGCAGGACAGCTCT	105.0	187
*nfkbia-3*	XM_021600117	AGAGTGGCCAATGTCGAAGTCT	TTGTGCGTCCAGTAACATATTATC	97.1	175
*il1b*	NM_001124347	GAGAGTGCTGTGGAAGAACATAT	GCAGCTCCATAGCCTCATTCAT	100.0	157
*cxcl8* (all variants)	AJ279069	CTGAGGGGATGAGTCTGAGAG	ATCTCCTGACCGCTCTTGCTC	105.1	169
*il4/13-1*	NM_001246341	CTGTCAGAGGAACTTCTGGAAAC	TCACCAAACGCGTCATTTTTCAC	101.7	131
*tgfb-1*	XM_021591332	ATCAGGGATGAACAAGCTGAGG	TTCGCACACAGCAACTCTCCG	72.0	161
*tgfb-2*	XM_021563342	CATTCCAAGGTGCTAGGTCTGT	TCTTGGGGGTCTTGCCGATGT	99.9	121
*socs1*	NM_001146166	ACGGATTCTGCGTCGGAAAATAT	ACACAGTTCCCTGGCATCCGT	104.4	92
*sod2*	XR_002474449	TCCCTGACCTGACCTACGAC	GGCCTCCTCCATTAAACCTC	100.1	201
*gpr84*	XM_021609929	AACTGAGGATGCTGGAGAACGT	GCACGCCGAAGTAGCGGTAG	100.0	96

To profile the infection-modulated *irak3* expression, we followed the principles of the 3Rs (37) for ethical use of animals and utilized samples from a previous experiment. Here, rainbow trout were infected with 1 × 10^7^ *Aeromonas salmonicida* ssp. *salmonicida* (approved animal experiment, LALLF M-V/TSD/7221.3-2.5-008/10.). The peritoneal injection has been previously described (22). Tissue samples from peripheral blood leucocytes (PBLs), head kidney and liver were used from four individuals at 0, 6, 12, and 24 h post-infection. The RNA was isolated from these samples in separate tubes using TRIzol (Invitrogen) and subsequently purified with the ISOLATE II RNA Micro Kit (Bioline). After cDNA synthesis, the concentrations of the individual cDNA aliquots were adjusted at 10 ng/5 μl. Samples were than measured using the Biomark/Fluidigm. To this end, cDNAs were individually pre-amplified in 10 cycles using the PreAmp Master Mix (Fluidigm) and subsequently treated with exonuclease I (New England BioLabs) to degrade single-stranded oligonucleotide primers. Finally, multiplex RT-qPCR was conducted using the 48.48 Fluidigm Gene Expression biochips, which were first primed in the MX IFC Controller (Fluidigm). Then they were loaded with the pre-amplified cDNA samples and eventually analyzed using the Biomark HD system with the manufacturer's thermal protocol “GE Fast 48 × 48 PCR+Melt v2.pcl.” EvaGreen fluorescence dyes (Bio-Rad) served as DNA-binding reporter molecules to allow for the quantification of the amplified target fragments.

The LightCycler-96 system (Roche) was used to quantify the expression of transcripts encoding inflammatory and inhibitory factors in stimulated and control CHSE-214 cells. The LightCycler protocol was optimized for a 12-μl-reaction volume using 6 μl SensiFAST SYBR No-ROX Mix (Bioline), 5 μl cDNA, and 1 μl primers. The qPCR program included an initial denaturation (95°C, 5 min.), followed by 40 cycles of denaturation (95°C, 5 s), annealing (60°C, 15 s), and elongation (72°C, 15 s), as well as the fluorescence measurement (72°C, 10 s).

All melting curves were analyzed individually to validate the absence of unspecific products. Amplicons were visualized on agarose gels in order to assess product size and quality. Crossing points (CP) between 5 and 35 cycles were considered detectable. The obtained real-time data were all normalized with a factor based on the geometric mean values from three potential reference genes. The qBase+ software (Biogazelle, Ghent University, Belgium) validated the suitability of the quantified reference genes, eukaryotic translation elongation factor *eef1a1* (38), ribosomal protein *rps5*, and 18S ribosomal RNA (39), for data normalization. Individual copy numbers were calculated based on external gene-specific standard curves (10^7^-10^3^ copies per 5 μl; *R*^2^ > 0.999).

## Results

From our 332 million RNA-sequencing reads from rainbow trout, we derived an ORF that encodes a 1920-nt comprising *irak3-*cDNA sequence. This trout sequence resembles reasonably well the human *IRAK3*-encoding sequence (~52% nucleotide-sequence identity) and the IRAK3 ortholog in orange-spotted grouper *Epinephelus coioides* (~63%), which represents the first published example of an *IRAK3* ortholog from teleost fish. The aa sequence derived from the conceptually translated cDNA shares >96% similarity with those from segments of the factor encoded by salmon and on average approximately 60% similarity with the ortholog factors from a wide range of fish species. The similarity of the Irak3-aa sequences is only about 40% in comparison to mammalian species.

### Two *irak3* Genes in Rainbow Trout Share Structural Similarities

**Figure 1:**
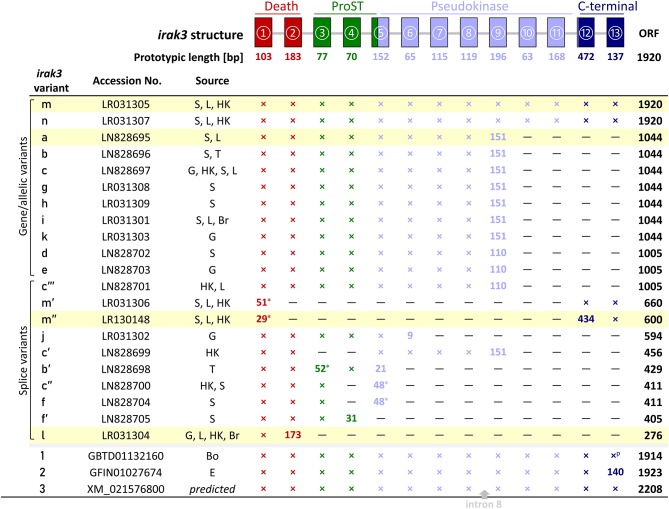
Representation of the different *irak3*-transcript lengths. The *irak3*-variants of trout are listed together with their accession numbers and their tissue-wise occurrence on the left. Tissues are abbreviated as follows: Bo, bone; Br, brain; E, unfertilized eggs; G, gills; HK, head kidney; L, liver; S, spleen; T, thymus. The genomic organization of the trout *irak3* gene is drawn above the scheme. Exons are represented by colored boxes (red, death domain; green, ProST region; light blue, pseudokinase domain; dark blue, C-terminal domain). Lines between exons represent introns. A cross indicates that the respective exon is present in the listed transcript variant; if the exon length differs from the prototypic length, the actual length is given (in nt); a superscript p denotes a partial 3′-end. The position where a fragment of intron 8 was inserted in the predicted *irak3* variant (3) is marked with a gray arrow. Asterisks indicate the possible usage of alternative splice acceptor/donor sites. The color of the characters denotes a particular protein domain. Fields highlighted in yellow indicate clones, which were functionally analyzed in later experiments (see Figures 5–7).

Inquiring into the gene structure, we used our RNA-seq-derived *irak3*-prototype sequence to search for *IRAK3* orthologs in the genome assemblies of rainbow trout (40–42) published by other groups and consortia. We identified two relevant entries for genomic trout sequences, (i) *irak3* (Gene ID: 101268966) on an unplaced scaffold (MSJN01015042, MSJN01016613, MSJN01023158) and (ii) *LOC110499741*/*irak3*-like (Gene ID: 110499741) on chromosome 21 (MSJN01004319). Comparing our cDNA sequence with these two different genomic deposits revealed that the latter, not contiguous sequence file consecutively comprises 13 exons that encode Irak3. Their lengths vary between 63 and 472 nt (excluding UTRs; Figure 1, upper scheme). The alignment of these exon sequences with the formerly mentioned three other genomic *irak3*-sequence files showed that they altogether harbor exons 1, 2, 5–7, and 9–13. The thus proven segmentation of the *irak3*-encoding gene from trout into 13 exons complies with a recent report on a homologous *irak3*-gene structure in grouper (28).

We compared the genomic sequences of both *irak3* genes from rainbow trout in order to understand if they stem from two different genes. The exon sequences are highly similar (>98% sequence identity). Due to large sequence gaps in both of the *irak3* genes, we conducted local alignments of comparable intronic fragments, which yielded identities between 90 and 99%. While this high degree of sequence similarity indicates their close relatedness, clear differences exist, and validate that they do belong to two different genes. For instance, the positions of the partially degenerated micro- or minisatellites within the intronic sequences appear to be conserved in both genes, albeit slightly altered (Table 2). Remarkably, we found repetitions of coding sequences in the introns of both *irak3* genes. For instance, the 5′-most 15 to 17 nt of exon 5 are repeated four times in intron 4, directly upstream of exon 5. Intron 7 of the *irak3* gene from trout features three tandem repetitions of exon 7 in a row, separated by a longer (>700 nt) and a shorter (314 nt) non-coding intronic sequence, each flanked by the canonical 5′-GT donor and 3′-AG acceptor dinucleotide splice sites. Similarly, a short fragment of exon 9 resides in intron 10, again flanked by core splice sites. Nonetheless, both deposits of genomic trout sequences for *irak3* and *LOC110499741*/*irak3*-like gene still contain gaps of unknown lengths in introns 1, 2, 3, 4, 11, and 12.

**Table 2:** Repetition of DNA elements (at least four-fold) within trout *irak3* genes (bases follow the standard IUPAC nucleotide codes).

**Accession number**	**Intron #**	**Motif sequence**	**Repetition**
***Irak3*** **(Gene ID: 101268966):**			
NW_018531519	1	TGAG	11
NW_018531519	1	AT	16
NW_018531519	1	AATSGAATCYGACACCGGASTCCCTAGTCNCTACWGTTA	7
NW_018536133	4	TACMACAGACT(GAT)GWTGTGTTSAGATGGTCCCTAGT	7
NW_018536133	4	GTAGAAACCACAGGCKTAGTGTTTTY(W)NNYYTRTTGCTMR	4
NW_018536133	6	TCTSTATGTCTAMCTACTCCAKTAATCCTCCATTTKAGATKGTT	14
NW_018536133	7	TACTCCASCATTTTAGATGGTTTCTSTATGTCTAMM	12
NW_018536133	between 7 and 12	KRACTACYTATAGAC(T)(R)	18
NW_018536133	between 7 and 12	TAACWNCCWMTARAN(T)(R)	29
***LOC110499741/irak3*****-like (Gene ID: 110499741):**			
MSJN01004319	1	AGTG	7
MSJN01004319	1	TA	19
MSJN01004319	1	ATACCATGGTACTAMTRKGGTACTTTCATKA	4
MSJN01004319	3	TG	4
MSJN01004319	4	TACATTAGATTRCTGAG(A)	25
MSJN01004319	4	GTAGAAACCACAGGCKTAGTGTTTT(T)YAYTCTGTTKCTAG	5
MSJN01004319	6	ATGTCTAMCTACTCCATTAATCCTCCATTTKAGATGGTTTCTGT	≥5
MSJN01004319	7	TGTTCCACAGACTGCATGACCAGGTGAGATATTCTATTCTMTACCCCTTACCTTCCCAAYGGATCWC	4
MSJN01004319	7	CTATTCTAGTTGTAKGTTGTGTTAC	9
MSJN01004319	8	TACCGGCTCAAACCCCTAGTCTATAACATCACAAGG	8
MSJN01004319	8	CAGTTAACCCACTGTTCCTAGRC	5
MSJN01004319	11	GT	4
MSJN01004319	11	RWAGCTWGGTGAATAYY	31
MSJN01004319	12	TAACTMCCTATAGAC(TR)	16

### More Than 20 *irak3*-Transcript Variants of Different Lengths Were Cloned From Rainbow Trout

**Figure 2:**
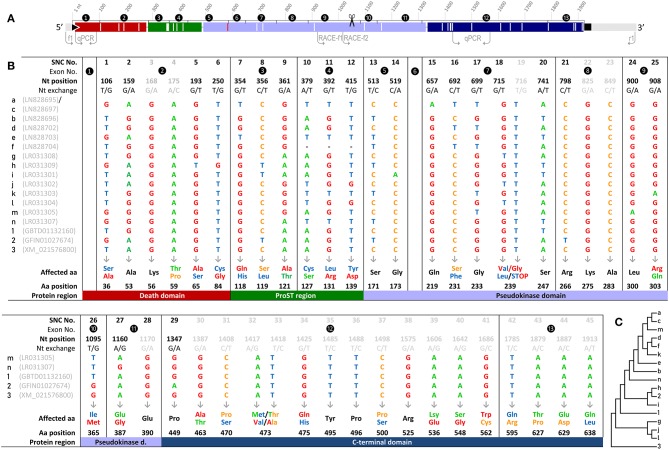
**(A)** Schematic illustration of the *irak3*-cDNA variants. The encoded protein domains are colored as in Figure 1 (upper panel) and drawn to scale. Flanking light gray lines indicate UTR sequences; the black triangle and square mark the start and stop of the ORF, respectively. Non-synonymous SNCs are represented by longitudinal white lines; the longitudinal red line indicates the point mutation that leads to the shortened variant *j*. Arrows indicate the locations of the primers used for fragment amplification and the 3′-RACE. The scissors symbol indicates the end of the truncated *irak3*-cDNAs. **(B)** List display of the 45 SNCs in trout Irak3-encoding cDNA sequences predicted by RNA-sequencing and those confirmed *via* cloning. The SNCs are numbered in the first row together with their exon-related occurrences and the exact positions in the ORF, with “1” assigned to the first nucleotide of the translational start triplet. Twenty-three of the SNCs were also confirmed by sequencing 100 randomly picked clones from batch-cloned *irak3* amplicons. SNCs not represented in that clone collection are shown in gray characters. Nucleotide variations and the affected aa residues are marked with the same colors (adenine, green; guanine, red; thymine, blue; cytosine, orange). Characteristic domains carrying the affected aa residues are indicated by differently shaded table fields. Note that variants *a* and *c* share the same SNCs, but bear an additional nt exchange at position 407 **(C)** Dendrogram of trout *irak3* cDNA variants. The bootstrap analysis of transcript relatedness involved 17 trout-specific nucleotide sequences (abbreviated as numbers and digits as indicated in **(B)** in the left column of the table).

We detected as many as 45 single nucleotide changes (SNCs) between nt positions 106 and 1,913 (numbering the first nucleotide of the translational start codon ATG as position 1) in our RNA-seq-derived *irak3*-consensus sequence (Figures 2A,B). To validate the variable nt positions in the *irak3* sequences of trout, we derived *irak3*-specific oligonucleotides (Figure 2A) from our prototype-*irak3* sequence for subsequent PCRs. Amplicons were cloned and more than 100 clones were sequenced. The cloned sequences validated 23 of the 45 SNCs (Figure 2B). Surprisingly, we found that the lengths of the encoded ORFs eventually differed significantly, ranging from 276 to 1,920 nt (Figure 1).

We named those varying *irak3* sequences in alphabetical order (a–m) according to their frequency in our clone collection and submitted them to the “European Nucleotide Archive” (ENA, project ID: **PRJEB29555**). The most abundant *irak3-*transcript variants encoded truncated versions of the prototypic 1,920-nt *irak3* ORF (Figure 1, table). They contained ORFs comprising 1,044 or 1,005 nt, subsequently termed as variants a (NCBI accession code: **LN828695**), b (**LN828696**), c (**LN828697**), d (**LN828702**), e (**LN828703**), g (**LR031308**), h (**LR031309**), i (**LR031301**), and k (**LR031303**). The variants m (**LR031305**) and n (**LR031307**) represent full-length *irak3* transcripts complying with two trout *irak3* sequences, which have been submitted to the ENA database (GBTD01132160 and GFIN01027674). Our two full-length sequences and those two derived from the database share an overall identity of >98%, differing in 20 positions due to those SNCs and a triplet insertion at the 3′ end of file GFIN01027674. In addition, the above-mentioned genome-derived *irak3*-like sequence LOC110499741 from file XM_021576800 features an ORF comprising 2,208 nt and is hence 288 nt longer than our cDNA-derived full-length *irak3* sequences. This additional 288-nt sequence stems from a microsatellite [(TACCGGCTCAAACCCCTAGTCTATAACATCACAAGG)_8_; c.f. Table 2] that had been incorporated into the cDNA sequence, since it does not interrupt the ORF in conceptual translation. A bootstrap test of our phylogenetic analysis (Figure 2C) only confirmed the strong similarity of variants a and c (98%) as well as of j and l (86%). The cDNA sequence derived from the *irak*3-like gene LOC110499741 was clearly separated from the other 14 variants (a–n), as well as from the sequences GBTD01132160 (termed here “1”) and GFIN01027674 (“2”) because of the insertion of the 288-nt microsatellite.

#### Some Truncated Transcripts Are Caused by Alternative Splicing

We found 10 shorter *irak3* transcript variants (Figure 1), which are most likely splice variants of the full-length factor m (m′, **LR031306**, m″, **LR130148**) or the truncated variants b (b′, **LN828698**), c (c′, **LN828699**; c″, **LN828700**; c^″′^ **LN828701**), f (**LN828704**, f′ (**LN828705**), j (**LR031302**), and l (**LR031304**). Most likely, two mechanisms caused the further size reduction of the full-length and truncated *irak3* transcripts. First, differential splicing exploited either the canonical splice sites GU and AG (variant c′) or alternative splice acceptor or donor sites located upstream of the commonly used dinucleotides GU (m′) or AG (b′, c″, f) shifted the ORF and provoked the incorporation of a pre-mature stop codon or shortened the exon size. Second, a point mutation (AAA → UAA, at position 591; variant j) introduced a stop codon and lead to the pre-mature termination of the ORF.

#### Some Truncated Transcripts Contain a Nonameric Sequence Absent in the Full-Length cDNA Sequence

**Figure 3:**
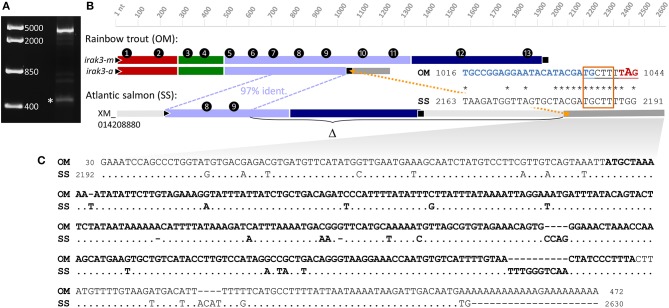
**(A)** 3′-RACE experiments using sense primers derived from exon 9 of the trout *irak3* gene (c.f. Figure 2A) produced two major bands of different length representing fragments of the truncated (marked with an asterisk) and the full-length transcript. DNA-marker bands are shown on the left. **(B)** Schematic comparison of the full-length (*irak3-m*) and truncated (*irak3-a*) transcripts of trout with the predicted Irak3-encoding fragment of salmon. Exons are numbered and sequences are drawn to scale; different colors indicate characteristic Irak3 domains as in Figures 1, 2. The homolog sequence shared by the coding *irak3* fragment of the truncated variant and the salmon sequence is indicated with a blue dotted line. The orange dotted line marks the position of the nonamer sequence motif (underlined) not found in the full-length transcript. The alignment of a section containing the nonamer-sequence in the truncated trout sequence (OM; LN828695) and in the salmon sequence (SS; XM_014208880) is shown on the right. Start and end nt positions of the fragments are indicated. The sequence of the RACE primer f2 is shown in blue. The potential accessory splice site is framed in orange; the stop codon of the truncated *irak3* transcripts is marked in red. **(C)** Alignment of the 5′-UTRs of the truncated *irak3* transcript from trout (OM; LR213462) and the predicted *irak3* sequence from salmon (SS; XM_014208880). Identical bases in the SS sequence are indicated by dots. The retroposon sequence is highlighted in bold.

The structural cause for the truncation of the *irak3* variants comprising the ORFs of 1,044 and 1,005 nt in length could not be explained based solely on the available DNA-sequence information. These truncated cDNA sequences feature a 9-nt-sequence motif at the 3′-end of their ORFs that is not found in the copies of the full-length cDNAs, or in the available genomic DNA sequences from trout. To elucidate the origin of the truncated transcript variants, we performed 3′-RACE experiments with the sense primer RACE-f1 annealing to the 5′-region of exon 9 and the nested primer RACE-f2 annealing further downstream on the same exon (Figure 2A). The resulting 3′-RACE sequence (**LR213462**, Figure 3A) did not match either the full-length *irak3-*cDNA sequences of trout or the substantial amount of intronic sequence information from the *irak3* and the *irak3*-like genes. It shares instead a high identity with an *irak3*-like sequence from Atlantic salmon (LOC106609858, XM_014208880). The trout 3′-RACE sequence matches from position 16 (located in exon 9) to the 3′ end of the salmon *irak3* UTR (positions 2,192–2,628) with >86% similarity (Figure 3B). Remarkably, comparing both sequences revealed that a point mutation introduced a translational stop codon in the truncated *irak3* variants from trout (Figure 3B, right side). The TGG triplet in the salmon sequence at positions 2,189–2,191 is converted into a TAG stop codon at positions 1,042–1,044 in the truncated *irak3* variants (a, b, b′, c, c′, c″, f, g, h, i, j, k, and l) from trout. Another noteworthy feature is a 275-nt long sequence residing in the 3′ UTR of both salmonid sequences (Figure 3C). Conceivably, this sequence stems from a retroposon element, since it is also present in many other genes from trout and salmon including such evolutionary unrelated genes as *specc1, orai1, eef1e1, anapc1*, and *morn3*. This mobile genetic element must have been inserted into the ancestral salmonid *irak3* gene, before the separation of the trout and salmon species.

### Irak3 Transcripts From Rainbow Trout Contain 45 Single Nucleotide Exchanges, but Only Three Different Alleles Were Found to Be Expressed in Individuals

The 45 identified SNCs in the *irak3*-cDNA sequences from rainbow trout are not randomly distributed across the coding sequence (Figures 2A,B). Rather, no exchanges were found in exons 1 and 6, whose coding sequences are strictly conserved. Moreover, exon 8 features only synonymous exchanges. The occurrence of the SNCs in the other exons does not follow an unambiguous pattern that would indicate any closer relatedness between the different *irak3* variants, as proven by our phylogenetic analysis (Figure 2C).

We tried to identify the minimal number of alleles from which the plethora of variants might possibly be encoded. To this end, we separately examined the *irak3* reads obtained from different tissues from each individual that was included in the RNA-seq analysis. We examined potential linkage groups formed by neighboring SNCs on the short 20–70-nt long reads and identified no more than three different SNC-linkage groups per individual. These individual linkage groups were expressed in all tissues analyzed. Hence, no tissue-specific linkage group was found, but SNC combinations did vary among the different individuals. Based on the assumption that one of these SNC combinations comes from the paternal allele and the other from the maternal allele, we conclude that the different alleles originated from (at least) two *irak3* genes.

Taken together with the structural analysis of the two highly segmented *irak3*-encoding genes, which revealed the presence of duplicated/multiple exon copies and/or exon fragments, it seems likely that the various *irak3* variants represent differentially spliced transcripts derived from only two *irak3* genes.

### Several Irak3 Variants From Trout Lack Function-Relevant Domains and Amino-Acid Residues

The full-length variants of *irak3* in rainbow trout encode 639-aa comprising proteins (m, **VDB38424**; n, **VDB38426**), which contain the following domains in accordance with the structural characteristics of IRAK-family members (2–5): (i) a death domain (aa positions 4–96), (ii) a ProST region (aa positions 94–161), (iii) a pseudokinase domain (aa positions 164–442) and (iv) the C-terminal region (aa positions 444–639). Twenty-nine of the 45 SNCs mentioned above (almost two-thirds of the SNCs) represent non-synonymous mutations that contribute to a striking diversity of Irak3 variants in rainbow trout. Remarkably, the relative abundance of non-synonymous exchanges is very low in the pseudokinase domain at only 0.6%, while it is significantly higher at in the ProST region (2.9%), followed by the C-terminal domain (2.0%) and the death domain (1.4%). Even more diversity of Irak3 factors is caused through the length reduction of several variants. The Irak3 variants a to k are shorter by at least 292 aa residues compared with the full-length factors m and n. The shortenings involve the deletion of about one third of the pseudokinase domain and the C-terminal domain (Figure 2A). In contrast, variants m′ and m″ contain the complete C-terminal domain and a part of the death domain, but lack the entire pseudokinase domain.

The death domain is encoded by the first two coding exons of the *irak3* and the *irak3*-like gene. Strikingly, the exon-1-encoded sequence is completely conserved in all *irak3* variants, except the splice variants m′ and m″ (Figure 1). The extremely short ORF of variant l (276 nt; Figure 1) only encodes an isolated death domain. The death domain of most other Irak3 variants is followed by the ProST region. This region contains 11 proline (16%), 15 serine (22%), and 4 threonine residues (6%) in a stretch of 69 aa residues. The relatively high abundance of these three aa residues corresponds approximately to those determined for all vertebrate IRAK proteins (43). Nevertheless, two of the vital ProST residues (Ser-119, Ser-127) are affected by base exchanges in some *irak3* variants (a, c, e, h, i, j, k, n; Figure 1A).

The pseudokinase domain covers approximately the central half of the full-length Irak3 aa sequence from trout. This domain harbors the IRAK3-characteristic residue Asn-292, which is present in all full-length and truncated Irak3 variants, but absent in most splice variants (m′, m″, b′, c″, f, f′, j, and l). The C-terminal domain is encoded by exons 12 and 13 and is therefore only present in the full-length factors m and n and their splice variants m′ and m″.

### Infection With *A. salmonicida* Modulates the Expression of *irak3* and Other Immunoregulatory Genes

**Figure 4:**
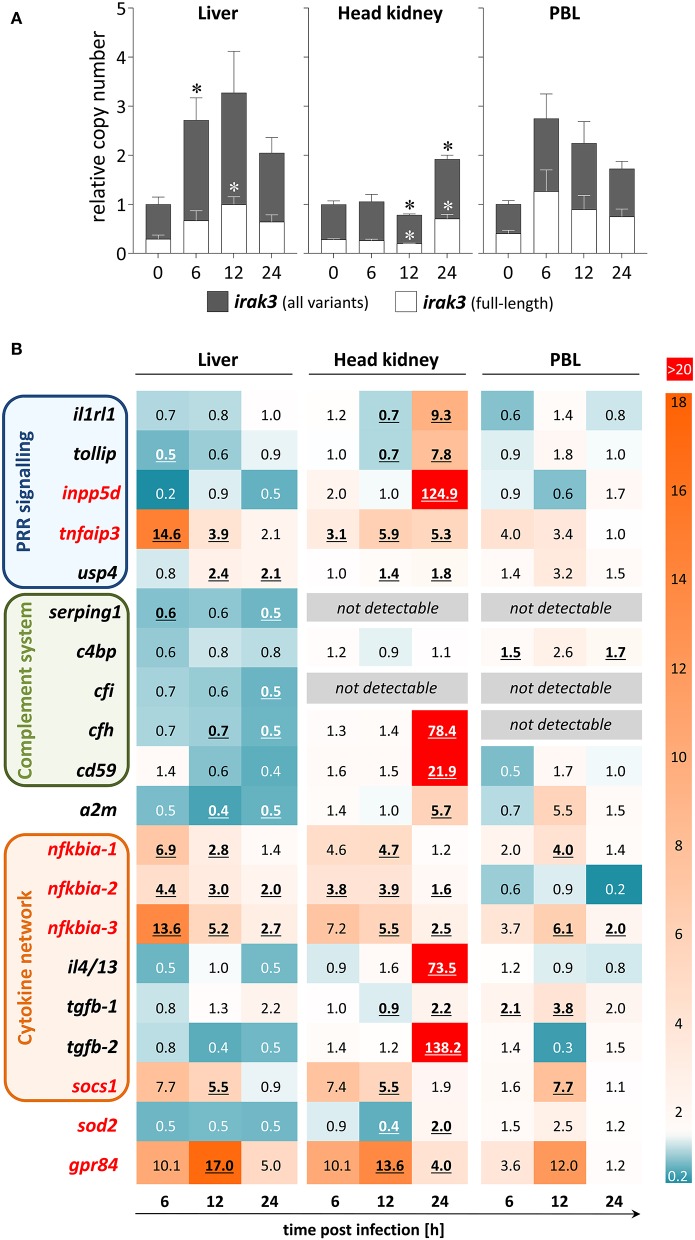
Expression profile of regulatory immune genes during *A.-salmonicida* infection of rainbow trout. **(A)** We used discriminating primer pairs to determine the abundance of all *irak3* variants in trout (filled columns) and the proportion of the full-length *irak3* transcripts (open columns) in the liver (left panel), head kidney (central), and PBLs (right) of four individuals at the various time points after infection relative to uninfected fish (0 h; set as 1.0). Significant copy-number changes (*p* < 0.05) are marked with an asterisk (^*^); error bars indicate SEM. **(B)** The HeatMap illustrates the averaged fold-change values (according to the legend on the right) of the mRNA concentrations measured in the liver (left panel), head kidney (central), and PBLs (right) at the time points after infection indicated below the scheme relative to controls (set as 1.0). The quantified transcripts are listed as gene symbols on the left and categorized according to their affiliation to immune processes; orthologs whose expression is IRAK3-dependent in mammals according to Zhou et al. (5) are marked in red. Significant copy-number changes (*p* < 0.05) are underlined. All expression values were normalized against the geometric means of the reference genes *eef1a1* and *rps5* as evaluated with the qBase+ software.

In healthy rainbow trout, the proportion of full-length transcripts within the entirety of all *irak3*-transcript variants varied in different tissues and cells: 29.1% (liver), 33.7% (head kidney), and 40.2% (PBLs) (Figure 4A). Hence, the transcripts that encode the full-length factor contribute much less than half of all *irak3* transcripts.

We used samples from a previous infection trial with live *A. salmonicida* pathogens (44) to determine the infection-related regulation of the expression of inflammation inhibitors. The copy numbers of all *irak3* variants were significantly elevated in the liver at 6 hpi and in the head kidney at 24 hpi (Figure 4A). The infection-related modulation of the *irak3*-transcript levels showed a significant ~2.7-fold increase in the liver and PBLs as early as 6 hpi and an approximate doubling of the transcript number was recorded in the head kidney, but only as late as 24 hpi. The ratio of full-length vs. truncated *irak3* variants was only slightly modulated during infection (<10% variation in each tissue/cell type).

The modulations of the expression levels of other inhibitor-encoding genes were also tissue-specific (Figure 4B). We detected in the head kidney very high transcript concentrations of numerous inhibitory factors (*il1rl1, tollip, inpp5d, tnfaip3, cfh, cd59, a2m, il4/13, tgfb-2, gpr84*) mostly at 24 hpi. In contrast, the expression of only a few regulators (*nfkbia, socs1*, and *gpr84*) increased in PBLs, especially at 12 hpi. In the liver, many genes were down-regulated, including complement-system regulators, some of which were not detectable in other cell/tissue types.

Certain genes were clearly upregulated across all cells/tissues and most time points. *Tnfaip3*/*a20* and the three *nfkbia* variants all significantly increased in the liver and head kidney at almost all times after infection, and considerable copy numbers were found in the PBLs as well. *Socs1* and *gpr84* were also strongly upregulated in all three cell/tissue types, but only at 12 hpi. Therefore, these data reveal that the *A.-salmonicida* infection triggered a broad variety of factors and mechanisms that counteract inflammation, validating that this infection trial was successful.

### Overexpression of *irak3*-a in Mammalian Cells Reduces Cell Viability

**Figure 5:**
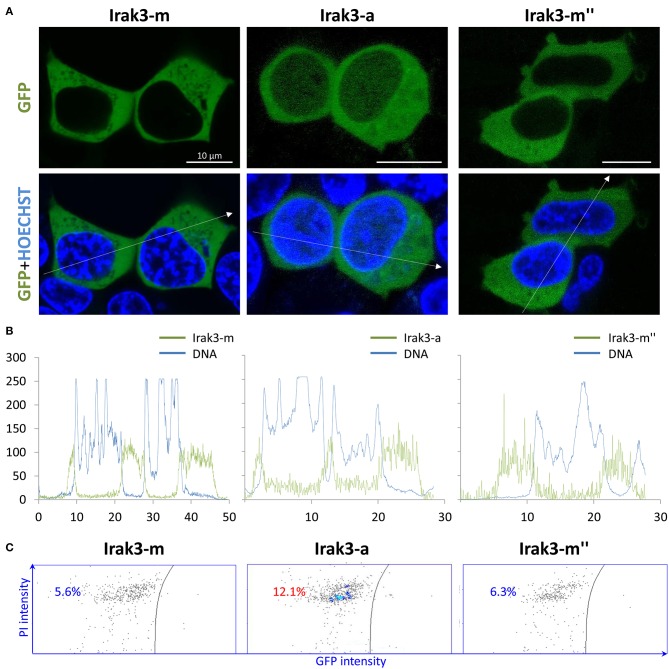
Overexpression of full-length factor Irak3 m and variants a, and m″ in HEK-293 cells. **(A)** Human HEK-293 cells were transfected with plasmids expressing the GFP-tagged Irak3 variants, the full-length factor m, truncated factor a, and splice variant m″. Cells were visualized 24 h after transfection with a confocal laser-scanning microscope. Nuclei were stained with Hoechst 33342 (blue). Scale bars represent 10 μm in all images. **(B)** Profile of fluorescence intensities (ordinate) recorded at specific locations (abscissa) across the cell following that path as indicated by the dotted arrows in 4A. **(C)** Live/dead assay of HEK-293 cells quantified the proportion of dead cells in the total cell count after the overexpression of full-length Irak3-m, truncated Irak3-a or spliced Irak3-m″ factors. The left part of each of the three dot plots shows the number of dead (PI-stained) GFP-negative cells and the right part shows the number of living GFP-positive (*irak3*-expressing) cells. One representative result is shown.

Previous investigations revealed that IRAK3 is equally distributed in the cytoplasm and nucleus of human cells and shifts toward the cytoplasm upon immune stimulation (45, 46). We wanted to see, whether this also applies to the ortholog from trout and whether the Irak3 variants might alter this pathogen-stimulated spatial redistribution of the factor. To this end, we transfected the full-length *irak3* or selected variants tagged with GFP into HEK-293 cells together with a plasmid expressing bovine *TLR2* (Figure 5A). Twenty-four hours after transfection, we observed that the full-length factor Irak3-m and its severely crippled splice variant m″ were almost uniformly distributed in the cytoplasm of the cells. The truncated Irak3-a, featuring the N-terminal half of the full-length factor and notably lacking the entire C-terminal domain, was visible both in the cytoplasm and in the nucleus (Figure 5B). This was seen in multiple transfections performed with different plasmid preparations. A stimulation of the cells with 10 ng/ml Pam_2_CSK_4_ did not change the localization (data not shown).

Strikingly, we found a higher proportion of rounded cells in those cultures that were transfected with the truncated Irak3-a variant (c.f. Figure 5A). Since the rounding up of otherwise fibroblast-like HEK-293 cells indicates cell death, we quantified the dead cells in cultures that were transfected with one of the three variants m, a, and m″. The fraction of dead cells doubled to >12% in cells transfected with *irak3-a* compared to non-transfected cells or cells transfected with *irak3-m* and -*m*″ (Figure 5C). The *in vivo* most abundantly expressed *irak3*-transcript variant appears to have a somewhat cytotoxic effect, at least if massively overexpressed.

### Full-Length Irak3-m and Truncated Variant a Increase Basal NF-κB Levels, but Do Not Reduce Stimulation-Induced NF-κB Levels in Mammalian Cells

**Figure 6:**
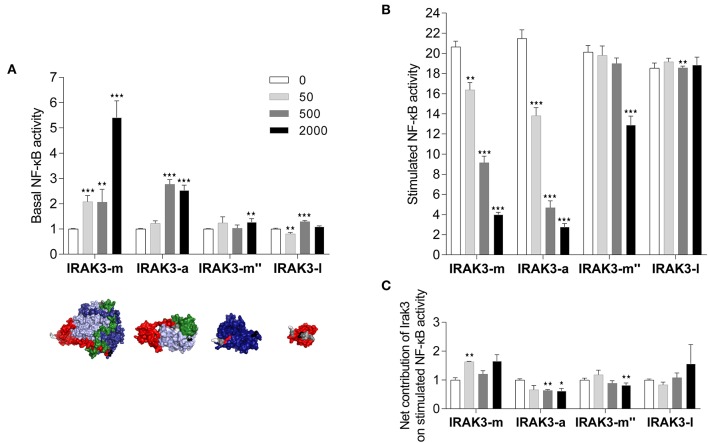
Reporter-gene analysis assessing the influence of Irak-3 factors on NF-κB activity in HEK-293 cells. HEK-293 cells were co-transfected with constructs expressing the bovine TLR2 and the mammalian ELAM-driven NF-κB-luciferase reporter together with increasing amounts of the plasmids (gray-scaled columns) that express the *irak3* variants m, a, m″, and l. **(A)** Fold-change values (ordinate) of the basal luciferase activity 24 h after transfection of *Irak3*-encoding plasmids, relative to the control (open columns) having received the empty plasmid only and no stimulation. The tertiary structures of the transfected *irak3* variants are displayed below the diagram. **(B,C)** Fold-change values of the luciferase activity after stimulation of TLR2 signaling with 10 ng/ml Pam_2_CSK_4_ and 100 ng/ml FSL-1 for 24 h, **(B)** relative to unstimulated control cells having been transfected with the same amount of the respective *irak3*-encoding plasmids, **(C)** relative to stimulated control cells transfected with the empty plasmid. Mean values from 2 or 3 independent experiments, each assayed in triplicate, are given. Statistical significance compared with the control group was assessed using one-way ANOVA (^*^p < 0.05; ^**^p < 0.01; ^***^p < 0.001).

In the next step, we investigated the influence of the Irak3 variants m, a, m″, and l (without GFP tag) on TLR2-induced NF-κB activation in mammalian HEK-293 cells. We transfected increasing amounts of constructs that express these factors into those cells. The co-transfection of an NF-κB-responsive luciferase-reporter gene allowed monitoring the NF-κB activity. Considering the effect of overexpressed Irak3 factors on the basal NF-κB activity without stimulating TLR2 signaling, we found that overexpressing the full-length factor m and the truncated factor a substantially increased the basal level of NF-κB activity in a dose-dependent fashion (Figure 6A), eventually to 5.4 ± 0.4 or 2.5 ± 0.2-fold (factors m and a, respectively) if the highest dose (2 μg of the plasmids) was used. In contrast, overexpression of the more severely shortened splice variants l and m″ induced only minor levels of active NF-κB.

Stimulating TLR2 signaling with the PAMPs Pam_2_CSK_4_ and FSL-1 in non-*irak3*-transfected HEK-293 cells increased the level of active NF-κB by 20.2 ± 0.4-fold compared with unstimulated non-*irak3*-transfected cells. Overexpressing the full-length factor m and the truncated variant a lowered the TLR2-signaling-induced degree of NF-κB activation down to 4.0 ± 0.2 or 2.8 ± 0.3-fold, respectively, compared to identically transfected, unstimulated controls. Splice variant l was basically ineffective (Figure 6B). Taking into account the stimulatory effect of the factors m and a on the basal NF-κB activity in the cells, it becomes clear that none of the transfected Irak3 variants substantially altered the ligand-dependent stimulation of NF-κB (Figure 6C). Altogether, the effect of the Irak3 variants on dampening the stimulated NF-κB activity was rather modest, not dose-dependent, and most likely independent from the properties of the transfected constructs.

We obtained almost congruent results when using heat-killed *E. coli* bacteria instead of PAMPs to stimulate HEK cells (data not shown). In addition, we also examined the effect of isoforms of the truncated *irak3* variants (g, h, k), but we did not observe any differences in modulating the levels of active NF-κB compared with Irak3 variant a (data not shown).

### Splice Variant irak3-m″, but Neither the Full-Length nor the Truncated Variants, Reduces the Expression of Pro-Inflammatory Interleukins in Salmonid Cells

**Figure 7:**
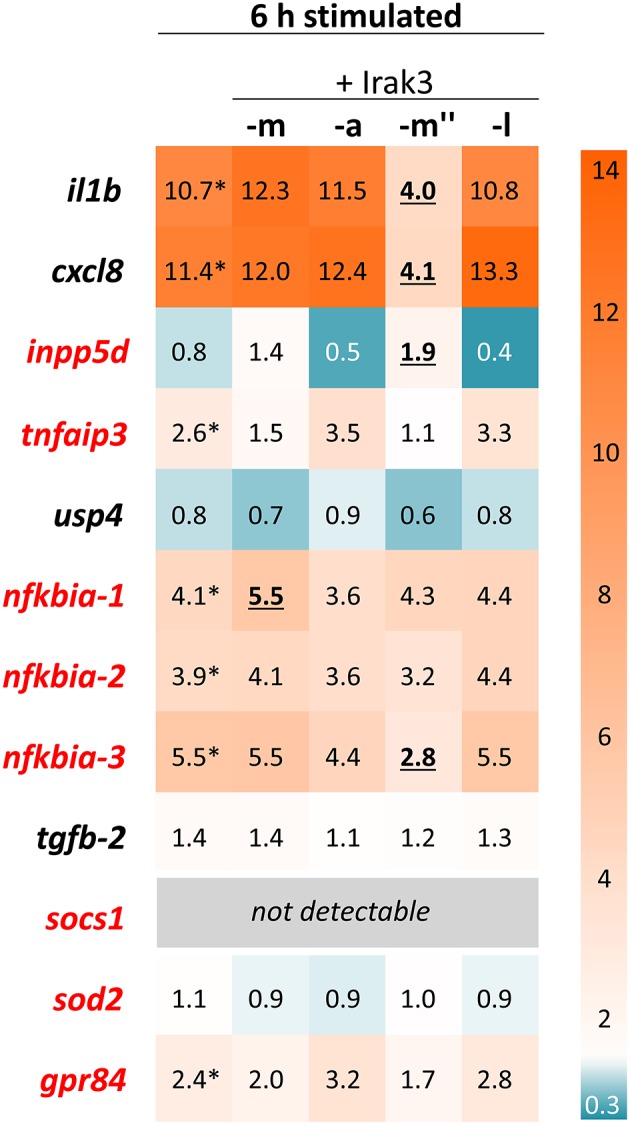
Expression profiling of stimulated Irak3-overexpressing CHSE-214 cells. The HeatMap illustrates the averaged fold-change values (according to the legend on the right) of the mRNA concentrations measured in CHSE cells 6 h after stimulation with 10 μg/ml poly (I:C) and 100 ng/ml flagellin, relative to unstimulated control cells (set as 1.0). The quantified transcripts are listed as gene symbols on the left; orthologs whose expression is IRAK3-dependent in mammals according to Zhou et al. (5) are highlighted in red. Significant copy-number changes (*p* < 0.05) of stimulated vs. non-stimulated untransfected cells are marked with asterisks; significant copy-number changes of stimulated non-transfected vs. transfected cells are underlined. All expression values were normalized against the geometric means of the reference genes *eef1a1* and *rps5*.

We examined the potentially different physiological significance of the prototypic Irak3 and its shorter variants. To this end, we overexpressed the full-length *irak3*, the truncated variant a and the splice variants m″ and l in the salmonid cell line CHSE-214 and quantified their influence on the transcript levels of selected immune genes (Figure 7). Transfected cells and non-transfected controls were stimulated with a mixture of the TLR3 ligand poly (I:C) and the TLR5 ligand flagellin for 6 h. qPCR analyses of *irak3*-transcript levels revealed that overexpression of the full-length *irak3* factor increased the basal *irak3*-transcript level in unstimulated cells by approximately 2,000-fold above the level of ~25 copies per ng of total RNA measured in non-transfected CHSE-214 cells.

Stimulating the non-transfected control cells for 6 h significantly increased the expression of the pro-inflammatory cytokines *il1b* and *cxcl8* by ~11-fold as well as the expression of the inflammation dampener *nfkbia-1–3* (3.9- to 5.5-fold), *tnfaip3* (2.6-fold), and *grp84* (2.4-fold) (Figure 7). Surprisingly, overexpression of neither the full-length *irak3* factor m, nor the truncated factors a and splice variant l caused any noteworthy changes in the expression of the selected indicator genes for monitoring inflammation. In stark contrast, splice variant m″ clearly and significantly decreased the transcript levels of *il1b* and *cxcl8* by a >5-fold, accompanied by a slight increase in *inpp5d* copies (~2-fold) over the levels found in stimulated but non-transfected cells. The 3-h stimulation yielded similar effects, albeit to a lesser extent (Supplementary Figure 1).

## Discussion

The interleukin-1-receptor-associated kinase 3 (IRAK3) was identified as a negative feedback regulator of inflammatory events (47, 48) and hence as a relevant factor for properly calibrating the immune response against infections. Its counterpart in the economically important salmonids had not yet been examined in more detail (1).

### At Least Two and Possibly More Genes Encode Irak3 in Rainbow Trout

At the outset of our study, we derived an *irak3*-consensus sequence from our sequence data obtained previously in an RNA-seq analysis of rainbow-trout-tissue samples. An alignment of the resulting consensus sequence with database entries of genomic DNA sequences suggested the existence of two different, yet closely related *irak3* genes that are encoded in rainbow-trout genome. Thirteen exons encode the full-length Irak3 factor in this salmonid species.

The detection of severely truncated cDNA sequences featuring a nonameric sequence, which is absent in both the full-length *irak3*-cDNA sequences and the two different genomic *irak3*-sequences, might suggest that these truncated variants are encoded by a third gene copy. However, we identified a highly similar counterpart to this nonamer in the 3′-UTR of an incomplete *irak3* transcript from Atlantic salmon. It might be plausible that the truncated transcripts from trout result from splicing a section of the 3′UTR to a degenerated copy of exon 9. This hypothetic copy may reside in an intronic region of the *irak3* gene similar to the fragments of exon 5, exon 7, and exon 9 within particular introns of the *irak3* genes. This could explain why the same nonameric sequence motif is found in two different transcripts featuring truncated exon-9 sequences of either 151-nt or 110-nt length. That assumption is supported by the fact that the respective nonameric sequence contains the motif TGCTT, which has previously been identified in an analysis of >20,000 human mRNAs as one of the 10 most frequent “accessory splicing signals” (49, 50).

In mammals, at least half of all full-length transcripts appear together with a splice variant (51). Splice variants also exist from *IRAK3* transcripts. For example, splice variant *IRAK3*Δ(2) is known in humans and *IRAK3*Δ(9−11) in mice (13). We also found spliced transcript variants that are clearly derived from the transcripts encoding the full-length or the truncated factor. In some cases, exons are spliced-out using the canonical splice-donor and acceptor dinucleotides flanking the enclosed intron. In other cases, the RNA splicing machinery apparently uses alternative more 5′-located splice-donor dinucleotides. This produces crippled exon sequences, which are incorporated into the resulting transcripts (b′, c″, f, m′, m″). Intriguingly, splicing at alternative, non-canonical splice-acceptor or -donor sites apparently produces an even larger proportion of differently shortened transcripts. We recently described a similar observation for the acute-phase gene *tcbp* coding for the trout C-polysaccharide binding protein (39). The usage of non-canonical splice sites may thus be a quite common phenomenon in teleost fish and salmonids in particular. Apparently, this species complex is still struggling with the consequences of the recent whole-genome duplication (41, 52) to sort out the most efficient gene copies from an excessive number.

### High Selection Pressure Rests on the Pseudokinase Domain of Irak3 From Rainbow Trout

Within the 1,920 nt ORF of the *irak3* consensus sequence, we detected 45 single-nucleotide changes. Hence, about every 40th base was found to be variable, potentially yielding more than 2^45^ = 3 × 10^13^ combinations. A recent report indicates that on average every 64th nucleotide position may be polymorphic in the pseudotetraploid genome of rainbow trout (42). Our study thus revealed an even more frequent occurrence of nucleotide exchanges in the *irak3* sequences of rainbow trout. The frequency of SNCs appears to be gene-specific, since the trout *irak4* paralog of *irak3*, for instance, revealed only six SNCs in a 1,425 nt sequence [Supplementary Figure 2; (22)].

Mapping the 29 non-synonymous SNCs on the full-length *irak3*-consensus sequence from rainbow trout illustrates that the pseudokinase domain is affected by the fewest aa-residue exchanges compared to the other three Irak3 domains. This pseudokinase domain, which is obviously under a higher selection pressure, discriminates the inflammation-supporting IRAK members−1 and−4 from the negatively regulating IRAK3 (3, 12). To our knowledge, there is no evidence yet that the pseudokinase domain of IRAK3 orthologs lacks phosphorylation activity. In general, the pseudokinase domain has so far received only little scientific attention. Therefore, we do not know much about the biological significance of exchanged aa residues within this domain.

The domain-mapping-of-disease-mutations database currently lists 15 non-synonymous SNPs in the <2 kb ORF of the human *IRAK3* gene. Several studies have proven significant associations between a particular SNP in one of the resulting IRAK3 variants and unbalanced immune reactions, including (i) asthma (53, 54), (ii) sepsis (55) and sepsis-induced acute lung injury (56), as well as (iii) a positive response to treatment in rheumatoid arthritis (57), and (iv) protection against *Bordetella pertussis* after vaccination (58). Additionally, certain SNPs of the human *IRAK3* gene are associated with the risk of cancer (59–61). Clearly, our study was not designed to analyze the physiological effects of SNPs/SNCs. It remains to be seen if any of these many Irak3 variants detected in our study are associated with a disease trait in rainbow trout.

### Irak3 Variants Differentially Activate Basal NF-κB Levels, but Not Their Final Level After Stimulation

We selected four major structural variants of Irak3 from rainbow trout to study and compare their functions *in vitro* in the human HEK-293-cell system. This cell line is a well-established “workhorse” for the heterologous expression of immune factors from fish (23, 36, 39, 62–64), especially for the reconstitution of the TLR-signaling cascade. The chosen Irak3 factors included the prototypic full-length Irak3-m factor (639 aa), the truncated Irak3-a variant lacking a part of the pseudokinase and the complete C-terminal domain (347 aa), and the splice variant Irak3-m″ lacking the ProST and the pseudokinase domain (199 aa). We were also curious to see, whether IRAK3-l, which consists only of a death domain (91 aa), would be functional. The HEK-293 cells were transfected with increasing amounts of plasmids that express either one out of the four selected Irak3 variants together with a plasmid expressing the bovine TLR2 and an NF-κB-responsive reporter vector. This experimental setup accounted for the well-explored fact that TLR signaling activates the NF-κB-transcription-factor complex (10) and should allow IRAK3 to counteract NF-κB-induced pro-inflammatory processes.

The overexpression of the full-length factor m and the truncated factor a from rainbow trout significantly increased basal NF-κB activity, while the shorter splice variants m″ and l were basically ineffective. Modulation of basal NF-κB activity was not necessarily expected, since overexpressing other regulatory factors of the TLR-signaling cascade, such as Il1rl1/St2 (36) or Irak4 from rainbow trout (22), left the level of basal NF-κB activity unchanged. The different modes of action of the full-length Irak3 factor m and its variants a, m″, and l also became evident after stimulating TLR2-transfected HEK-293 cells with relevant PAMPs. Noteworthy, the summation of basal and induced NF-κB-induction folds yielded, in effect, similar values for the final NF-κB-activity levels after stimulation. It was rather unexpected that the overexpression of full-length *irak3-m* did not alter the final level of NF-κB activity after stimulation with TLR2 ligands, given that Irak3 has often been proven to be a dampener of TLR-dependent inflammation (12, 47, 48, 65). However, a survey of the pertinent literature showed that NF-κB levels after pathogen-dependent stimulation has not often been used as a read-out system. The first analysis on human IRAK3 (alias IRAK-M) 20 years ago demonstrated that overexpressed IRAK3 increased basal NF-κB-activity levels without stimulation (3). This is generally in line with subsequent studies (4, 28) and also with our findings. Many other reports on IRAK3 function did not examine its direct impact upon the TLR-mediated activation of NF-κB activity, but instead used different read-out systems to validate the inflammation-dampening effects of IRAK3, such as the level of cytokine-gene expression (13, 66, 67). We show here that Irak3 from rainbow trout does not directly target NF-κB to inhibit inflammatory processes.

Notwithstanding, our overexpression experiment in HEK-293 cells proved that the truncated Irak3 variant a increases the basal level of activated NF-κB almost as efficient as the full-length variant, although it lacks the entire C-terminal domain. This domain is apparently not involved in the mechanism that regulates the observed effect. Parallel microscopic observations detected a, perhaps unrelated to NF-κB activation, difference between full-length and the truncated Irak3 variants. We found that the truncated variant Irak3-a increased cell death, in contrast to the full-length variant. Although the death domain is a well-explored structural requirement for the execution of apoptotic processes (68), there is no evidence, to our knowledge, that IRAK3 directly affects apoptosis in cells. For this reason, we can only speculate whether the physiological expression of the C-terminally truncated Irak3-a variant has a toxic effect on cells *in vivo*.

### The Irak3 Splice Variant m″ May Act as a Scavenger Molecule of TLR Signaling

Our studies conducted in the salmonid CHSE-214 model cells focused on the Irak3-dependent modulation of immune-gene expression after stimulating the TLR axis. We found that the stimulation of immune-gene expression is largely uninfluenced by the overexpression of either the full-length factor *irak3*-m or its variants a or l. We note in this regard that previous studies on the function of the mammalian IRAK3 have been performed primarily on macrophages and lung epithelial cells (13, 71). An embryonic cell such as CHSE-214 may not provide all the factors required for the correct functioning of Irak3. Moreover, we found a low endogenous expression level of *irak3* in CSHE-214 cells, but it is remarkable that the additional, eventually 2,000-fold increased concentration of the transcripts encoding the Irak3 factors m, a, and l did not influence at all the induced immune-gene expression. Only overexpression of the splice variant m″ significantly lowered the expression of *il1b* and *cxcl8*. The stimulated induction of these two proinflammatory cytokines is known to heavily depend on the activation of the TLR-signaling cascade. The assumption that Irak3 splice variant m″ hinders TLR signaling is supported by the fact that its overexpression also led to decreased *nfkbia-3* expression. It is known from mammals that activated TLR signaling concomitantly induces the expression of NF-κB-inhibitory factors such as NFKBIA (alias IκBα). This negative feedback loop conceivably prevents excessive NF-κB activation (69, 70). The Irak3 splice variant m″ consists almost exclusively of the C-terminal domain. It lacks parts of the death domain and the entire ProST region which is required in the mammalian ortholog for interacting with IRAK4 and activating NF-κB (3–5). This particular architecture of m″ might prevent its integration (*via* TIR-TIR interactions) into the TLR-associated myddosome and could explain why only high concentrations of m″ reduce the NF-κB activity levels (c.f. section Irak3 Variants Differentially Activate Basal NF-κB Levels, But not Their Final Level After Stimulation). Our 3D-model analysis shows that m″ exposes the C-terminal domain more prominently than the full-length factor Irak3-m. This domain contains the motif Pro-X-Glu-X-X(aromatic/acidic residue), which is crucial for the interaction with the downstream signaling factor TRAF6 (72) and is well-conserved in the full-length Irak3 factor and its splice variant m″. Hence, Irak3-m″ might perform its inhibitory function on the expression of *il1b* and *cxcl8* by scavenging TRAF6 in a highly efficient way. This could, in turn, reduce the intensity of TLR-mediated NF-κB activation.

It is poorly understood in the mammalian ortholog, how the shortening of the full-length factor impacts the function of IRAK3, as encoded by the respective splice variants. IRAK2 factors might perhaps serve as a paradigm. There are four isoforms of IRAK2 in mouse and two alternatively spliced factors act as inhibitory factors (73), while the other two support the TLR-signal transfer in a positive manner (74). This example illustrates how the splicing of a particular mRNA transcript may reverse the basic function of the original protein and extend its functional spectrum with either additional or even antagonizing aspects.

### Expression Profiling Provides no Indications That any of the *irak3* Variants From Trout Induces the Expression of Other Immune Regulators

In parallel to *irak3*, we profiled the expression of a broader set of immune inhibitors in infected rainbow trout with focus on those genes whose expressions are considered IRAK3-dependent. Previous publications on mammalian models reported that IRAK3 induces the expression of genes coding for SOCS, INPP5D (SHIP1), TNFAIP3 (A20), NFKBIA (IκBα), SOD2, and GPR84 (5, 15, 17). In fact, we observed an early upregulation of *irak3* transcripts concomitant with increased levels of *inpp5d, tnfaip3, nfkbia-2* and *-3, sod2*, and *gpr84* in the head kidney and/or liver. However, our overexpression studies on the salmonid cell line CHSE-214 did not provide any evidence for the involvement of Irak3 factors in stimulating the expression of specific immune inhibitors. Only the overexpression of variant m″ led to an increase in the expression of *inpp5d*.

Using discriminating primers, we determined the abundances of all *irak3* transcripts and the full-length *irak3* transcripts during the infection of trout with *A. salmonicida*. Within 24 hpi, *irak3* transcript levels increased significantly both in the liver and head kidney, but not in PBLs. These results complement our previous observation that *irak3*-transcript levels were elevated at 72 hpi with *A. salmonicida* in the gills of rainbow trout (75). The infection-related increase in the expression of *irak3* has previously been reported in embryonic zebrafish (27).

In summary, our *in-vivo* analyses of selected Irak3 factors from rainbow trout revealed that both, the full-length and the truncated Irak3 factor contain the structural requirements that allow modulating the activation status of NF-κB. The splice variant Irak3-m″ from trout limits the expression of inflammatory cytokines. However, we found no evidence that Irak3 from trout actually induces the expression of other immune regulators in contrast to its mammalian counterpart.

## Conclusions

The mammalian IRAK3 factor controls not only the various TLR-dependent signaling cascades but also the signal transduction downstream of the interleukin-1 receptor (IL1R1). While only one full-length and one splice variant of human IRAK3 have been reported, we found more than 20 *irak3* cDNA variants in the salmonid fish rainbow trout, which probably stem from two duplicated genes. It is possible that the presence of these several *irak3*-transcript variants is only a collateral and functionally insignificant consequence of the polyploidization history of salmonids (41, 52). Nevertheless, these multiple Irak3 variants may represent not only sheer abundance, but could cover different and/or complementing tasks. The individual *irak3* variants from trout could, for instance, integrate specifically into the different cascades mediated by IL1R1 and more than a dozen TLRs (44, 76–79) present in pseudo-tetraploid salmonids. Although our study suggests that the trout Irak3 factors do not interfere with the stimulation-dependent NF-κB activation, we found that only, and perhaps significantly, a specific Irak3-splice variant eventually downregulates the expression of certain cytokine genes. This demonstrates that the great structural diversity of the irak3 factors from trout may also translate into some functional diversity.

## Data Availability Statement

The novel datasets generated for this study are included in the manuscript/Supplementary Files. All *irak3*-cDNA sequences have been submitted to the “European Nucleotide Archive” (project ID: PRJEB29555).

## Ethics Statement

The animal experiment has been approved by the Landesamt für Landwirtschaft, Lebensmittelsicherheit und Fischerei, Mecklenburg-Vorpommern, Germany; LALLF M-V/TSD/7221.3-2.5-008/10.

## Author Contributions

H-MS and AR designed the research. TG and H-MS supervised the project. MV and TG performed RNA-seq experiments. SH, JK, and AR cloned Irak3 variants and transfected cells. AR, H-MS, MV, SH, JK, and TG analyzed sequences. HR carried out confocal microscopy and vitality tests. AR and SH performed reporter-gene analysis. AR carried out qPCR experiments. AR and H-MS wrote the paper. All authors commented on the manuscript.

### Conflict of Interest

The authors declare that the research was conducted in the absence of any commercial or financial relationships that could be construed as a potential conflict of interest.

## Abbreviations

Aa: amino acid
GFP: green-fluorescent protein
hpi: hours post-infection
INPP5D: inositol polyphosphate-5-phosphatase D
IRAK: interleukin-1 receptor-associated kinase
LPS: lipopolysaccharide
NF-κB: nuclear factor “kappa-light-chain-enhancer” of activated B-cells
NFKBIA: NF-κB inhibitor subunit alpha
nt: nucleotide(s)
PI: propidium iodide
qRT-PCR: quantitative reverse transcriptase polymerase chain reaction
SNC: single nucleotide change
SNP: single-nucleotide polymorphism
SOCS: suppressor of cytokine signaling
TIR: Toll/interleukin-1 receptor resistance
TLR: Toll-like receptor
TNFAIP3: tumor necrosis factor alpha-induced protein 3
TRAF6: tumor necrosis factor alpha receptor–associated factor 6
UTR: untranslated region.

## References

[1] Rebl A Goldammer T. Under control: the innate immunity of fish from the inhibitors' perspective. Fish Shellfish Immunol. (2018) 77:328–49. 10.1016/j.fsi.2018.04.016 29631025

[2] Gosu V Basith S Durai P Choi S. Molecular evolution and structural features of IRAK family members. PLoS ONE. (2012) 7:e49771. 10.1371/journal.pone.0049771 23166766 PMC3498205

[3] Wesche H Gao X Li XX Kirschning CJ Stark GR Cao ZD. IRAK-M is a novel member of the pelle/interleukin-1 receptor-associated kinase (IRAK) family. J Biol Chem. (1999) 274:19403–10. 10383454 10.1074/jbc.274.27.19403

[4] Du J Nicolaes GA Kruijswijk D Versloot M van der Poll T van 't Veer C. The structure function of the death domain of human IRAK-M. Cell Commun Signal. (2014) 12:77. 10.1186/s12964-014-0077-3 25481771 PMC4273448

[5] Zhou H Yu M Fukuda K Im J Yao P Cui W. IRAK-M mediates Toll-like receptor/IL-1R-induced NFκB activation and cytokine production. EMBO J. (2013) 32:583–96. 10.1038/emboj.2013.2 23376919 PMC3579143

[6] Cormican P Lloyd AT Downing T Connell SJ Bradley D O'Farrelly C. The avian Toll-Like receptor pathway–subtle differences amidst general conformity. Dev Comp Immunol. (2009) 33:967–73. 10.1016/j.dci.2009.04.001 19539094

[7] Lin SC Lo YC Wu H. Helical assembly in the MyD88-IRAK4-IRAK2 complex in TLR/IL-1R signalling. Nature. (2010) 465:885–90. 10.1038/nature09121 20485341 PMC2888693

[8] Nie L Cai S-Y Shao J-Z Chen J. Toll-like receptors, associated biological roles, and signaling networks in non-mammals. Front Immunol. (2018) 9:1523. 10.3389/fimmu.2018.01523 30034391 PMC6043800

[9] Vijay K. Toll-like receptors in immunity and inflammatory diseases: past, present, and future. Int Immunopharmacol. (2018) 59:391–412. 10.1016/j.intimp.2018.03.002 29730580 PMC7106078

[10] O'Neill LAJ Golenbock D Bowie AG. The history of Toll-like receptors - redefining innate immunity. Nat Rev Immunol. (2013) 13:453–60. 10.1038/nri3446 23681101

[11] Karin M Lin A. NF-kappaB at the crossroads of life and death. Nat Immunol. (2002) 3:221–7. 10.1038/ni0302-221 11875461

[12] Janssens S Beyaert R. Functional diversity and regulation of different interleukin-1 receptor-associated kinase (IRAK) family members. Mol Cell. (2003) 11:293–302. 10.1016/S1097-2765(03)00053-4 12620219

[13] Kobayashi K Hernandez LD Galan JE Janeway CA Jr Medzhitov R Flavell RA. IRAK-M is a negative regulator of Toll-like receptor signaling. Cell. (2002) 110:191–202. 10.1016/s0092-8674(02)00827-9 12150927

[14] Hassan F Islam S Tumurkhuu G Dagvadorj J Naiki Y Komatsu T. Involvement of interleukin-1 receptor-associated kinase (IRAK)-M in toll-like receptor (TLR) 7-mediated tolerance in RAW 264.7 macrophage-like cells. Cell Immunol. (2009) 256:99–103. 10.1016/j.cellimm.2009.01.013 19251253

[15] van 't Veer C van den Pangaart PS van Zoelen MAD de Kruif M Birjmohun RS Stroes ES Induction of IRAK-M is associated with lipopolysaccharide tolerance in a human endotoxemia model. J Immunol. (2007) 179:7110–20. 10.4049/jimmunol.179.10.7110 17982103

[16] Liu Z-J Yan L-N Li X-H Xu F-L Chen X-F You H-B. Up-regulation of IRAK-M is essential for endotoxin tolerance induced by a low dose of lipopolysaccharide in Kupffer cells. J Surg Res. (2008) 150:34–39. 10.1016/j.jss.2007.12.759 18533191

[17] Xiong Y Medvedev AE. Induction of endotoxin tolerance *in vivo* inhibits activation of IRAK4 and increases negative regulators IRAK-M, SHIP-1, and A20. J Leukoc Biol. (2011) 90:1141–8. 10.1189/jlb.0611273 21934070 PMC3236548

[18] Zhou Y Xia Q Wang X Fu S. Endotoxin tolerant dendritic cells suppress inflammatory responses in splenocytes via interleukin-1 receptor associated kinase (IRAK)-M and programmed death-ligand 1 (PDL-1). Med Sci Monit. (2018) 24:4798–806. 10.12659/MSM.908242 29995830 PMC6069485

[19] Lyn-Kew K Rich E Zeng X Wen H Kunkel SL Newstead MW. IRAK-M regulates chromatin remodeling in lung macrophages during experimental sepsis. PLoS ONE. (2010) 5:e11145. 10.1371/journal.pone.0011145 20585389 PMC2886833

[20] Günthner R Kumar VRS Lorenz G Anders H-J Lech M Pattern-recognition receptor signaling regulator mRNA expression in humans and mice, and in transient inflammation or progressive fibrosis. Int J Mol Sci. (2013) 14:18124–47. 10.3390/ijms140918124 24009023 PMC3794773

[21] Rebl A Goldammer T Fischer U Köllner B Seyfert H-M. Characterization of two key molecules of teleost innate immunity from rainbow trout (*Oncorhynchus mykiss*): MyD88 and SAA. Vet Immunol Immunopathol. (2009) 131:122–6. 10.1016/j.vetimm.2009.03.006 19362743

[22] Brietzke A Goldammer T Rebl H Korytář T Köllner B Yang W. Characterization of the interleukin 1 receptor-associated kinase 4 (IRAK4)-encoding gene in salmonid fish: the functional copy is rearranged in Oncorhynchus mykiss and that factor can impair TLR signaling in mammalian cells. Fish Shellfish Immunol. (2014) 36:206–14. 10.1016/j.fsi.2013.11.005 24239597

[23] Rebl A Rebl H Liu S Goldammer T Seyfert H-M. Salmonid Tollip and MyD88 factors can functionally replace their mammalian orthologues in TLR-mediated trout SAA promoter activation. Dev Comp Immunol. (2011) 35:81–7. 10.1016/j.dci.2010.08.012 20813127

[24] Huang R Lv J Luo D Liao L Zhu Z Wang Y. Identification, characterization and the interaction of Tollip and IRAK-1 in grass carp (Ctenopharyngodon idellus). Fish Shellfish Immunol. (2012) 33:459–67. 10.1016/j.fsi.2012.05.025 22659441

[25] Zhang C-Z Yin Z-X He W Chen W-J Luo Y-W Lu Q-X Weng S-P Yu X-Q He J. Cloning of IRAK1 and its upregulation in symptomatic mandarin fish infected with ISKNV. Biochem Biophys Res Commun. (2009) 383:298–302. 10.1016/j.bbrc.2009.03.137 19336221 PMC7092954

[26] Stein C Caccamo M Laird G Leptin M. Conservation and divergence of gene families encoding components of innate immune response systems in zebrafish. Genome Biol. (2007) 8:R251. 10.1186/gb-2007-8-11-r251 18039395 PMC2258186

[27] Stockhammer OW Zakrzewska A Hegedûs Z Spaink HP Meijer AH. Transcriptome profiling and functional analyses of the zebrafish embryonic innate immune response to Salmonella infection. J Immunol. (2009) 182:5641–53. 10.4049/jimmunol.0900082 19380811

[28] Li Y-W Han R Wang J-L Yang M Dan X-M Li A-X. Molecular identification and functional characterization of IRAK-3 from a teleost fish, the orange-spotted grouper (*Epinephelus coioides*). Fish Shellfish Immunol. (2018) 81:383–9. 10.1016/j.fsi.2018.07.029 30010020

[29] Goldammer T Rebl A Brunner RM Köbis J Verleih M Borchel A Global Transcriptome Analyses in Two Different Selected Rainbow Trout Strains for Development of Molecular Biomarkers Determining Fish Welfare. Aquaculture Europe 2015 Rotterdam. Available online at: https://www.was.org/easonline/AbstractDetail.aspx?i=4152 (accessed October 21, 2015).

[30] Golosova O Henderson R Vaskin Y Gabrielian A Grekhov G Nagarajan V. Unipro UGENE NGS pipelines and components for variant calling, RNA-seq and ChIP-seq data analyses. PeerJ. (2014) 2:e644. 10.7717/peerj.644 25392756 PMC4226638

[31] Mello B Estimating TimeTrees with MEGA and the TimeTree resource. Mol Biol Evol. (2018) 35:2334–42. 10.1093/molbev/msy133 29931306

[32] Kelley LA Sternberg MJE. Protein structure prediction on the Web: a case study using the Phyre server. Nat Protoc. (2009) 4:363–71. 10.1038/nprot.2009.2 19247286

[33] Sayle RA Milner-White EJ. RASMOL: biomolecular graphics for all. Trends Biochem Sci. (1995) 20:374. 7482707 10.1016/s0968-0004(00)89080-5

[34] Yang W Zerbe H Petzl W Brunner RM Gunther J Draing C. Bovine TLR2 and TLR4 properly transduce signals from *Staphylococcus aureus* and *E. coli*, but S. aureus fails to both activate NF-kappaB in mammary epithelial cells and to quickly induce TNFalpha and interleukin-8 (CXCL8) expression in the udder. MolImmunol. (2008) 45:1385–97. 10.1016/j.molimm.2007.09.004 17936907

[35] Shaw G Morse S Ararat M Graham FL. Preferential transformation of human neuronal cells by human adenoviruses and the origin of HEK 293 cells. FASEB J. (2002) 16:869–71. 10.1096/fj.01-0995fje 11967234

[36] Rebl A Rebl H Köbis JM Goldammer T Seyfert H-M. ST2 from rainbow trout quenches TLR signalling, localises at the nuclear membrane and allows the nuclear translocation of MYD88. Dev Comp Immunol. (2017) 67:139–52. 10.1016/j.dci.2016.10.009 27776995

[37] Flecknell P. Replacement, reduction and refinement. ALTEX. (2002) 19:73–8. Available online at: https://www.altex.org/index.php/altex/article/view/1106 12098013

[38] Bowers RM Lapatra SE Dhar AK. Detection and quantitation of infectious pancreatic necrosis virus by real-time reverse transcriptase-polymerase chain reaction using lethal and non-lethal tissue sampling. J Virol Methods. (2008) 147:226–34. 10.1016/j.jviromet.2007.09.003 17996958

[39] Köbis JM Rebl H Goldammer T Rebl A. Multiple gene and transcript variants encoding trout C-polysaccharide binding proteins are differentially but strongly induced after infection with *Aeromonas salmonicida*. Fish Shellfish Immunol. (2017) 60: 509–19. 10.1016/j.fsi.2016.11.021 27836722

[40] Palti Y Genet C Gao G Hu Y You FM Boussaha M. A second generation integrated map of the rainbow trout (*Oncorhynchus mykiss*) genome: analysis of conserved synteny with model fish genomes. Mar Biotechnol. (2012) 14:343–57. 10.1007/s10126-011-9418-z 22101344

[41] Berthelot C Brunet F Chalopin D Juanchich A Bernard M Noël B. The rainbow trout genome provides novel insights into evolution after whole-genome duplication in vertebrates. Nat Commun. (2014) 5:3657. 10.1038/ncomms4657 24755649 PMC4071752

[42] Gao G Nome T Pearse DE Moen T Naish KA Thorgaard GH. A new single nucleotide polymorphism database for rainbow trout generated through whole genome resequencing. Front Genet. (2018) 9:147. 10.3389/fgene.2018.00147 29740479 PMC5928233

[43] Kollewe C Mackensen A-C Neumann D Knop J Cao P Li S. Sequential autophosphorylation steps in the interleukin-1 receptor-associated kinase-1 regulate its availability as an adapter in interleukin-1 signaling. J Biol Chem. (2004) 279:5227–36. 10.1074/jbc.M309251200 14625308

[44] Brietzke A Korytář T Jaros J Köllner B Goldammer T Seyfert H-M. *Aeromonas salmonicida* infection only moderately regulates expression of factors contributing to Toll-like receptor signaling but massively activates the cellular and humoral branches of innate immunity in rainbow trout (*Oncorhynchus mykiss*). J Immunol Res. (2015) 2015:901015. 10.1155/2015/901015 26266270 PMC4525466

[45] Udgata A Qureshi R Mukhopadhyay S. Transduction of functionally contrasting signals by two mycobacterial ppe proteins downstream of TLR2 receptors. J Immunol. (2016) 197:1776–87. 10.4049/jimmunol.1501816 27481848

[46] Su J Xie Q Wilson I Li L. Differential regulation and role of interleukin-1 receptor associated kinase-M in innate immunity signaling. Cell Signal. (2007) 19:1596–601. 10.1016/j.cellsig.2007.02.009 17379480 PMC1978187

[47] Flannery S Bowie AG. The interleukin-1 receptor-associated kinases: critical regulators of innate immune signalling. Biochem Pharmacol. (2010) 80:1981–91. 10.1016/j.bcp.2010.06.020 20599782

[48] Rothschild DE McDaniel DK Ringel-Scaia VM Allen IC Modulating inflammation through the negative regulation of NF-κB signaling. J Leukoc Biol. (2018) 103:1131–50. 10.1002/JLB.3MIR0817-346RRR PMC6135699 29389019

[49] Suyama M Harrington ED Vinokourova S von Knebel Doeberitz M Ohara O Bork P. A network of conserved co-occurring motifs for the regulation of alternative splicing. Nucleic Acids Res. (2010) 38:7916–26. 10.1093/nar/gkq705 20702423 PMC3001076

[50] Voelker RB Berglund JA. A comprehensive computational characterization of conserved mammalian intronic sequences reveals conserved motifs associated with constitutive and alternative splicing. Genome Res. (2007) 17:1023–33. 10.1101/gr.6017807 17525134 PMC1899113

[51] Modrek B Lee C. A genomic view of alternative splicing. Nat Genet. (2002) 30:13–9. 10.1038/ng0102-13 11753382

[52] Macqueen DJ Johnston IA. A well-constrained estimate for the timing of the salmonid whole genome duplication reveals major decoupling from species diversification. Proc R Soc B Biol Sci. (2014) 281:20132881. 10.1098/rspb.2013.2881 24452024 PMC3906940

[53] Balaci L Spada MC Olla N Sole G Loddo L Anedda F. IRAK-M is involved in the pathogenesis of early-onset persistent asthma. Am J Hum Genet. (2007) 80:1103–14. 10.1086/518259 17503328 PMC1867098

[54] Pino-Yanes M Sánchez-Machín I Cumplido J Figueroa J Torres-Galván MJ González R. IL-1 receptor–associated kinase 3 gene (IRAK3) variants associate with asthma in a replication study in the Spanish population. J Allergy Clin Immunol. (2012) 129:573–5.e10. 10.1016/j.jaci.2011.10.001 22070913

[55] Dong G Gong J Li J Luo Y Li Z Li P. Association between gene polymorphisms of IRAK-M and the susceptibility of sepsis. Inflammation. (2013) 36:1087–93. 10.1007/s10753-013-9641-z 23588345

[56] Pino-Yanes M Ma S-F Sun X Tejera P Corrales A Blanco J. Interleukin-1 receptor–associated kinase 3 gene associates with susceptibility to acute lung injury. Am J Respir Cell Mol Biol. (2011) 45:740–5. 10.1165/rcmb.2010-0292OC 21297081 PMC3265218

[57] Sode J Vogel U Bank S Andersen PS Hetland ML Locht H. Confirmation of an IRAK3 polymorphism as a genetic marker predicting response to anti-TNF treatment in rheumatoid arthritis. Pharmacogenomics J. (2018) 18:81–6. 10.1038/tpj.2016.66 27698401

[58] Kimman TG Banus S Reijmerink N Reimerink J Stelma FF Koppelman GH. Association of interacting genes in the Toll-like receptor signaling pathway and the antibody response to pertussis vaccination. PLoS ONE. (2008) 3:e3665. 10.1371/journal.pone.0003665 18987746 PMC2573957

[59] Rajaraman P Brenner AV Butler MA Wang SS Pfeiffer RM Ruder AM. Common variation in genes related to innate immunity and risk of adult glioma. Cancer Epidemiol Biomarkers Prev. (2009) 18:1651–8. 10.1158/1055-9965.EPI-08-1041 19423540 PMC2771723

[60] Bhatnagar R Dabholkar J Saranath D. Genome-wide disease association study in chewing tobacco associated oral cancers. Oral Oncol. (2012) 48:831–5. 10.1016/j.oraloncology.2012.03.007 22503698

[61] Greenman C Stephens P Smith R Dalgliesh GL Hunter C Bignell G. Patterns of somatic mutation in human cancer genomes. Nature. (2007) 446:153–8. 10.1038/nature05610 17344846 PMC2712719

[62] Pietretti D Vera-Jimenez NII Hoole D Wiegertjes GFF. Oxidative burst and nitric oxide responses in carp macrophages induced by zymosan, MacroGard® and selective dectin-1 agonists suggest recognition by multiple pattern recognition receptors. Fish Shellfish Immunol. (2013) 35:847–57. 10.1016/j.fsi.2013.06.022 23831551

[63] Skjaeveland I Iliev DB Strandskog G Jørgensen JB. Identification and characterization of TLR8 and MyD88 homologs in Atlantic salmon (*Salmo salar*). Dev Comp Immunol. (2009) 33:1011–7. 10.1016/j.dci.2009.04.007 19422846

[64] Brietzke A Arnemo M Gjøen T Rebl H Korytář T Goldammer T. Structurally diverse genes encode Tlr2 in rainbow trout: The conserved receptor cannot be stimulated by classical ligands to activate NF-κB *in vitro*. Dev Comp Immunol. (2016) 54:75–88. 10.1016/j.dci.2015.08.012 26348603

[65] Liew FY Xu D Brint EK O'Neill LAJ. Negative regulation of toll-like receptor-mediated immune responses. NatRevImmunol. (2005) 5:446–58. 10.1038/nri1630 15928677

[66] Steiger S Kumar SV Honarpisheh M Lorenz G Günthner R Romoli S. Immunomodulatory molecule IRAK-M balances macrophage polarization and determines macrophage responses during renal fibrosis. J Immunol. (2017) 199:1440–52. 10.4049/jimmunol.1601982 28701510

[67] Zhang M Chen W Zhou W Bai Y Gao J. Critical role of IRAK-M in regulating antigen-induced airway inflammation. Am J Respir Cell Mol Biol. (2017) 57:547–59. 10.1165/rcmb.2016-0370OC 28665693 PMC6943878

[68] Weber CH Vincenz C. The death domain superfamily: a tale of two interfaces? Trends Biochem. (2001) 26:475–81. 10.1016/S0968-0004(01)01905-3 11504623

[69] Ghosh S Hayden MS. New regulators of NF-kappaB in inflammation. Nat Rev Immunol. (2008) 8:837–48. 10.1038/nri2423 18927578

[70] Hinz M Scheidereit C. The IκB kinase complex in NF-κB regulation and beyond. EMBO Rep. (2014) 15:46–61. 10.1002/embr.201337983 24375677 PMC4303448

[71] Hoogerwerf JJ van der Windt GJW Blok DC Hoogendijk AJ De Vos AF van 't Veer C. Interleukin-1 receptor-associated kinase M-deficient mice demonstrate an improved host defense during Gram-negative pneumonia. Mol Med. (2012) 18:1067–75. 10.2119/molmed.2011.00450 22729155 PMC3475335

[72] Ye H Arron JR Lamothe B Cirilli M Kobayashi T Shevde NK. Distinct molecular mechanism for initiating TRAF6 signalling. Nature. (2002) 418:443–7. 10.1038/nature00888 12140561

[73] Hardy MP O'Neill LA. The murine IRAK2 gene encodes four alternatively spliced isoforms, two of which are inhibitory. J Biol Chem. (2004) 279:27699–708. 10.1074/jbc.M403068200 15082713

[74] Muzio M Ni J Feng P Dixit VM. IRAK (Pelle) family member IRAK-2 and MyD88 as proximal mediators of IL-1 signaling. Science. (1997) 278:1612–5. 9374458 10.1126/science.278.5343.1612

[75] Rebl A Korytář T Köbis JM Verleih M Krasnov A Jaros J. Transcriptome profiling reveals insight into distinct immune responses to *Aeromonas salmonicida* in gill of two rainbow trout strains. Mar Biotechnol. (2014) 16:333–48. 10.1007/s10126-013-9552-x 24122123

[76] Altmann S Korytář T Kaczmarzyk D Nipkow M Kühn C Goldammer T. Toll-like receptors in maraena whitefish: evolutionary relationship among salmonid fishes and patterns of response to Aeromonas salmonicida. (2016) 54:391–401. 10.1016/j.fsi.2016.04.125 27131902

[77] Arnemo M Kavaliauskis A Gjøen T. Effects of TLR agonists and viral infection on cytokine and TLR expression in Atlantic salmon (*Salmo salar*). Dev Comp Immunol. (2014) 46:139–45. 10.1016/j.dci.2014.03.023 24736205

[78] Lee PT Zou J Holland JW Martin SAM Collet B Kanellos T. Identification and characterisation of TLR18-21 genes in Atlantic salmon (*Salmo salar*). Fish Shellfish Immunol. (2014) 41:549–59. 10.1016/j.fsi.2014.10.006 25450999

[79] Lee PT Zou J Holland JW Martin SAM Kanellos T Secombes CJ. Identification and characterization of TLR7, TLR8a2, TLR8b1 and TLR8b2 genes in Atlantic salmon (*Salmo salar*). Dev Comp Immunol. (2013) 41:295–305. 10.1016/j.dci.2013.05.013 23747412

