# Supplementary material for: At Least Two Genes Encode Many Variants of Irak3 in Rainbow Trout, but Neither the Full-Length Factor Nor Its Variants Interfere Directly With the TLR-Mediated Stimulation of Inflammation
Source: Front Immunol. 2019 Sep 20;10:2246. doi: 10.3389/fimmu.2019.02246 (PMC6763605; doi:10.3389/fimmu.2019.02246)
Supplement: Supplementary file 1 [file Data_Sheet_1.docx]

Supplementary Material

## Supplementary Figures

.
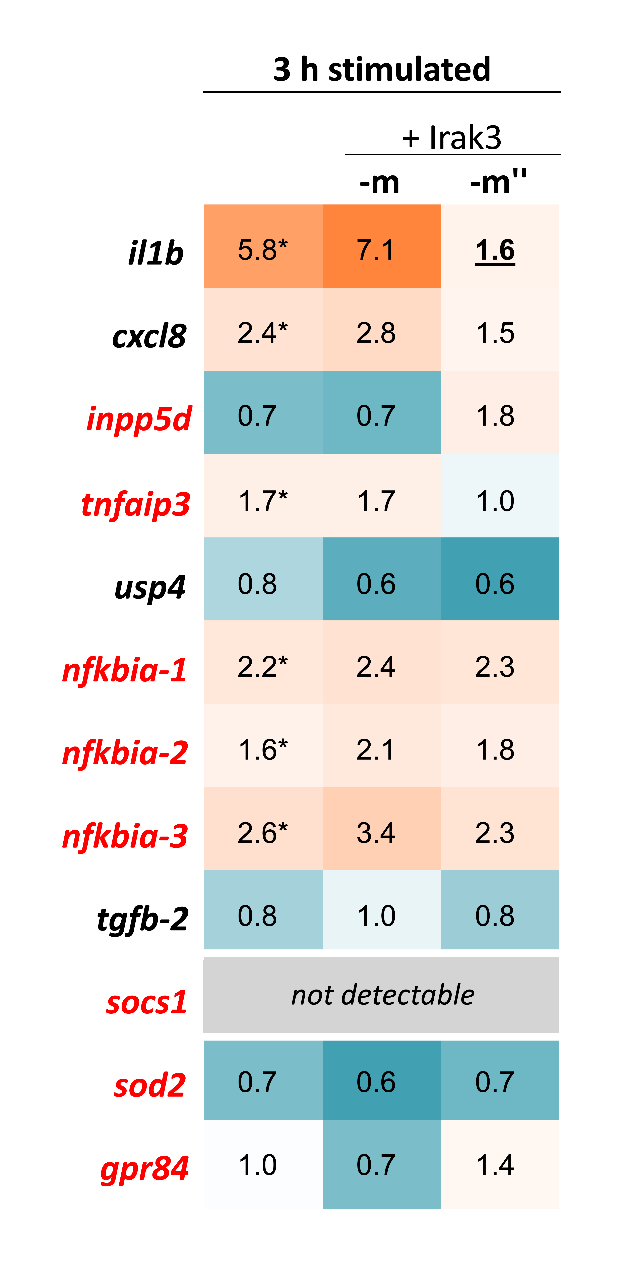


**Supplementary Figure 1.** Expression profiling of stimulated Irak3-expressing CHSE-214 cells. The HeatMap illustrates the averaged fold-change values of the mRNA concentrations measured in CHSE cells 3 h after stimulation with 10 µg/ml poly (I:C) and 100 ng/ml flagellin, relative to unstimulated control cells (set as 1.0). The quantified transcripts are listed as gene symbols on the left. Significant copy-number changes (p < 0.05) of stimulated versus non-stimulated untransfected cells are marked with asterisks; significant copy-number changes of stimulated non-transfected versus transfected cells are underlined. All expression values were normalised against the geometric mean of the reference genes *eef1a1* and *rps5*.

## Supplementary Figures


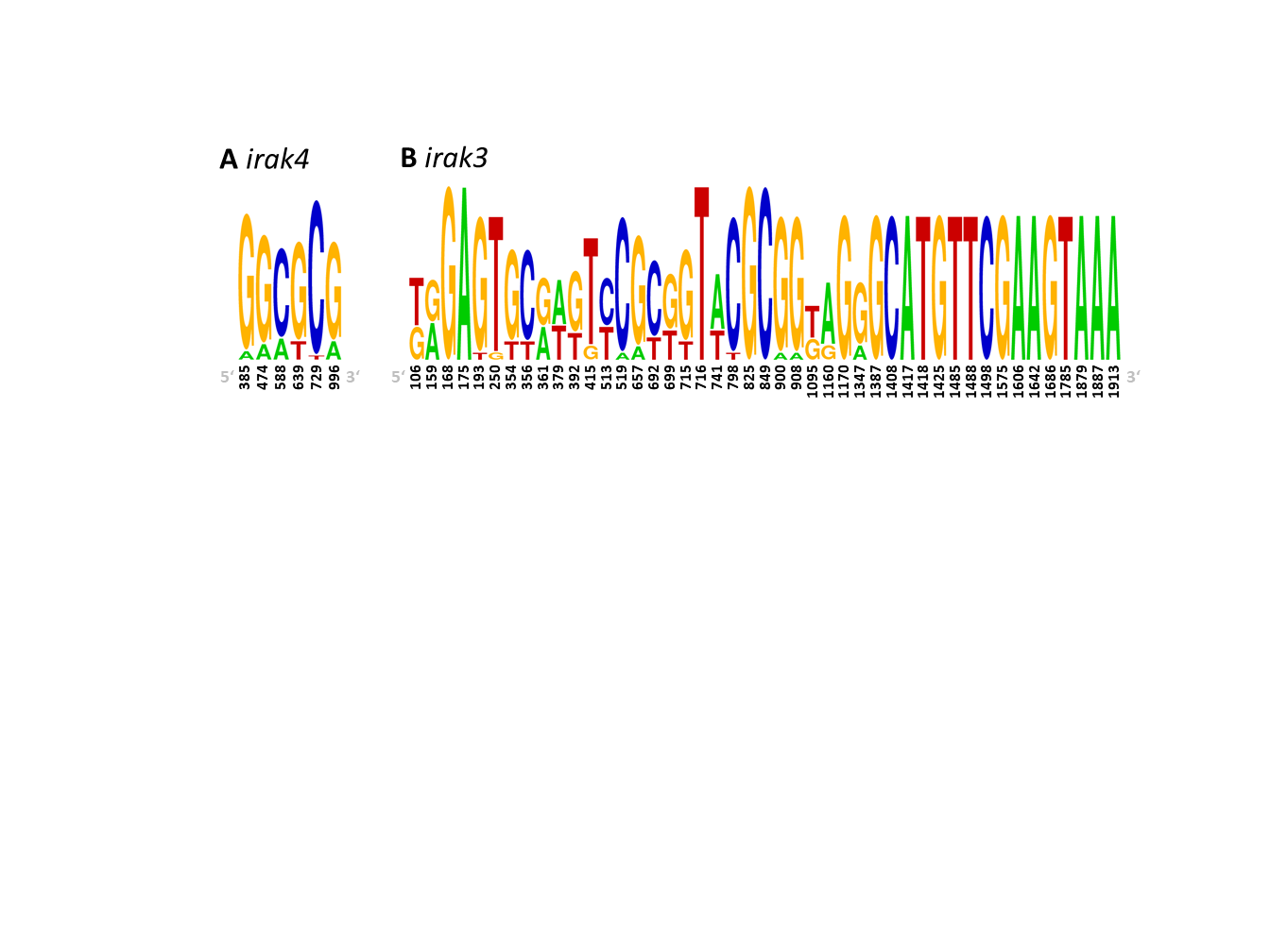


**Suppl. Fig. 2**: WebLogo 3 (<http://weblogo.threeplusone.com/>) representation of identified SNCs in coding sequences of the rainbow-trout genes (**A**) *irak4* and (**B**) *irak3*. The frequency of a base is expressed in the size of its symbol. The SNC position is given below the scheme.
